# Expression levels of long non-coding RNAs are prognostic for AML outcome

**DOI:** 10.1186/s13045-018-0596-2

**Published:** 2018-04-07

**Authors:** Arvind Singh Mer, Johan Lindberg, Christer Nilsson, Daniel Klevebring, Mei Wang, Henrik Grönberg, Soren Lehmann, Mattias Rantalainen

**Affiliations:** 1 Department of Medical Epidemiology and Biostatistics, Karolinska Institutet, Nobels Vag 12A, SE-17177 Stockholm, Sweden; 2 Department of Medical Epidemiology and Biostatistics, Science for Life Laboratory, Karolinska Institutet, Nobels Vag 12A, SE-17177 Stockholm, Sweden; 3 Hematology Centre, Karolinska University Hospital and Karolinska Institute, Huddinge, Stockholm Sweden; 4 Department of Medical Sciences, Uppsala University, Uppsala, Sweden

**Keywords:** lncRNA, Acute myeloid leukemia, Prognosis, Molecular subtype

## Abstract

**Background:**

Long non-coding RNA (lncRNA) expression has been implicated in a range of molecular mechanisms that are central in cancer. However, lncRNA expression has not yet been comprehensively characterized in acute myeloid leukemia (AML). Here, we assess to what extent lncRNA expression is prognostic of AML patient overall survival (OS) and determine if there are indications of lncRNA-based molecular subtypes of AML.

**Methods:**

We performed RNA sequencing of 274 intensively treated AML patients in a Swedish cohort and quantified lncRNA expression. Univariate and multivariate time-to-event analysis was applied to determine association between individual lncRNAs with OS. Unsupervised statistical learning was applied to ascertain if lncRNA-based molecular subtypes exist and are prognostic.

**Results:**

Thirty-three individual lncRNAs were found to be associated with OS (adjusted *p* value < 0.05). We established four distinct molecular subtypes based on lncRNA expression using a consensus clustering approach. LncRNA-based subtypes were found to stratify patients into groups with prognostic information (*p* value < 0.05). Subsequently, lncRNA expression-based subtypes were validated in an independent patient cohort (TCGA-AML). LncRNA subtypes could not be directly explained by any of the recurrent cytogenetic or mutational aberrations, although associations with some of the established genetic and clinical factors were found, including mutations in *NPM1*, *TP53*, and *FLT3*.

**Conclusion:**

LncRNA expression-based four subtypes, discovered in this study, are reproducible and can effectively stratify AML patients. LncRNA expression profiling can provide valuable information for improved risk stratification of AML patients.

**Electronic supplementary material:**

The online version of this article (10.1186/s13045-018-0596-2) contains supplementary material, which is available to authorized users.

## Background

Acute myeloid leukemia (AML) is a heterogeneous disease on both the molecular- and phenotypic level, caused by malignant transformation of hematopoietic progenitor cells. During pre-leukemic evolution and disease progression, affected hematopoietic cells gradually accumulate a range of molecular alterations, including somatic mutations, cytogenetic abnormalities, epigenetic alterations, and transcriptomic changes [1, 2]. Numerous recurrent point mutations, epigenetic changes, and cytogenetic abnormalities have been identified through next generation sequencing technology [1, 3]. Cytogenetics together with mutation status of *NPM1*, *CEBPA*, and *FLT3* internal tandem duplications (FLT3-ITD) form the basis of the European LeukemiaNet (ELN) risk classification system [4], which provides means for risk stratification of AML patients. However, almost half of patients are classified into the intermediate risk group. Further improvements of the risk stratification of AML patients would provide the potential for improved therapy decisions.

LncRNAs are defined as RNA molecules longer than 200 nucleotides that are transcribed while not protein coding. It has been estimated that more than 58,000 lncRNAs are encoded in the human genome [5, 6]. LncRNAs are involved in a multitude of biological processes that are central in tumorigenesis and progression of cancer, including cell cycle regulation, proliferation, apoptosis, migration, and genomic stability [5, 7]. LncRNAs have multiple modes of action, including involvement in controlling chromatin condensation, regulation of transcription, regulation of RNA splicing, controlling RNA stability, and promoting or inhibiting translation of mRNAs to proteins [8].

Most large-scale genomic analyses of cancer patient data have focused on the protein coding region of the genome. However, estimates from the ENCODE study suggest that up to 75% of the human genome gets transcribed into RNA, whereas only about 3% of the human genome is protein coding [9, 10]. LncRNAs are a group of non-coding RNAs that have several recent discoveries linked to cancer [11–13]. For example, *HOX* transcript antisense intergenic RNA (HOTAIR) is known to act as an epigenetic regulator in breast and colorectal cancer [14–16]. Several other lncRNAs are known to play a functional role as oncogenes or tumor suppressors and have clear prognostic potential [14, 17]. Multiple studies have highlighted the role of lncRNA in hematopoietic cellular development and malignancies. In T cell acute lymphoblastic leukemia (T-ALL), the lncRNA LUNAR1 (leukemia-induced non-coding activator RNA) promotes cell growth via enhanced *IGF1R* expression [18]. The IRAIN lncRNA, located within *IGF1R* locus, directly interacts with the *IGF1R* promotor [19]. IRAIN is shown to be downregulated in leukemia cell lines and in high-risk AML patients. Garzon et al. [7] have previously reported lncRNA expression results from a study consisting of cytogenetically normal acute myeloid leukemia (CN-AML) patients using a custom microarray platform for lncRNA expression profiling, with a focus on assessing association with routine clinical phenotypes and mutations. In that study, lncRNAs were reported to be associated with recurrent mutations in several genes in CN-AML patients, including *NPM1*, *CEBPA*, *IDH2*, *ASXL1*, and *RUNX1*, and FLT3-ITD [7, 20]. LncRNA expression has previously also been shown to be associated with treatment response and survival in several other cancer types [5, 21–23].

Despite growing evidence for the potential importance of lncRNAs as prognostic and diagnostic markers across a multitude of cancers, including AML, lncRNA expression in AML has not been comprehensively characterized to date with a focus on ascertaining the potential presence of prognostic lncRNA-based AML subtypes. In this study, we applied whole-transcriptome RNA-sequencing (RNA-seq) with the aim to identify prognostic lncRNAs, to define novel lncRNA-based AML subtypes and to ascertain their prognostic value and relevance for risk stratification of AML patients. Furthermore, novel lncRNA expression-based subtypes were validated in independent patient cohort.

## Results

**Fig. 1:**
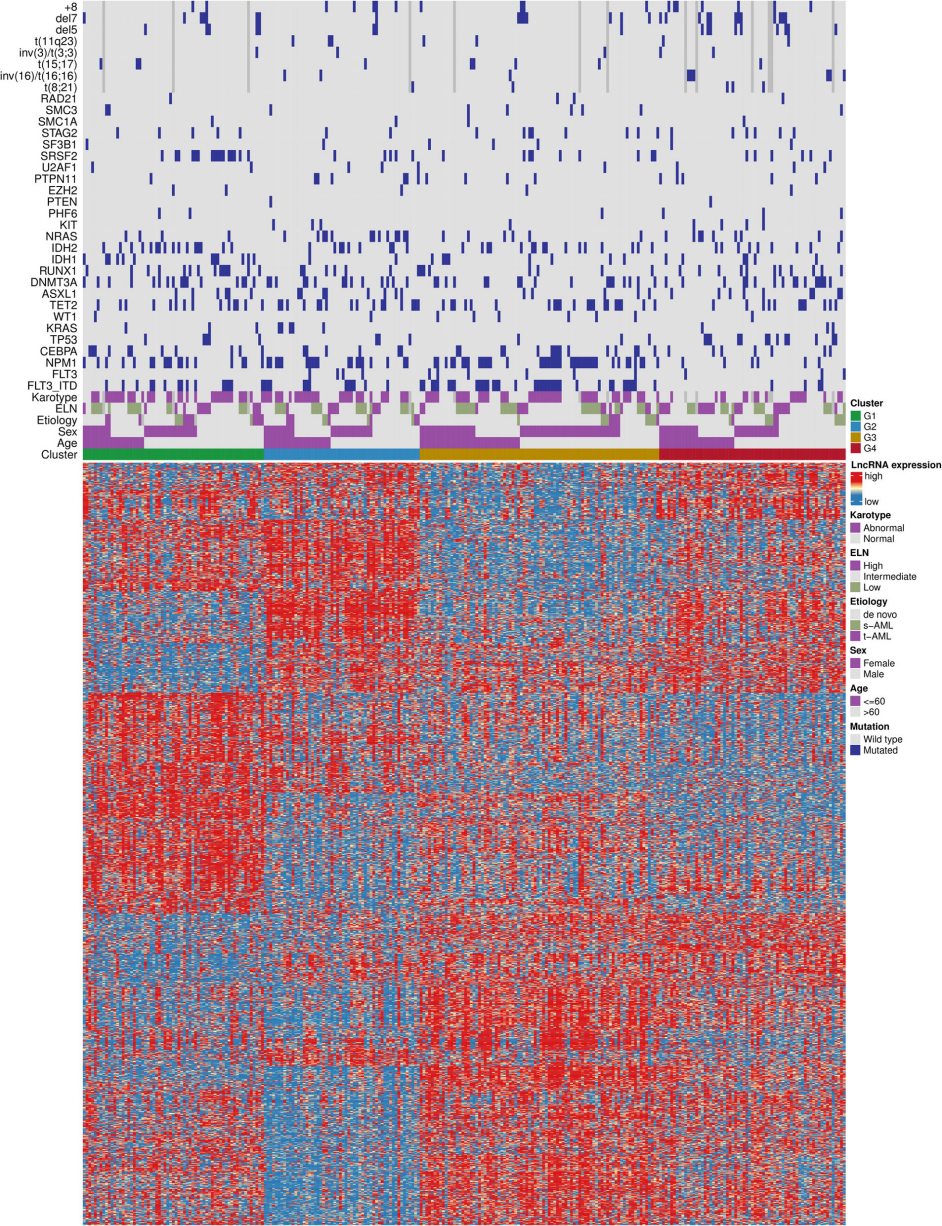
LncRNA expression patterns for four lncRNA-based novel AML subtypes together with clinicopathological factors, cytogenetic risk classification, mutation, and karyotype status. FLT3 represents FLT3-TKD

We applied RNA sequencing to characterize lncRNA expression in 274 intensively treated AML patients from the Clinseq-AML cohort (see the “Methods” section). The detailed characteristics of the Clinseq-AML cohort are shown in Table 1. LncRNAs were annotated using the MiTranscriptome database [6]. Using the consensus cluster [24] approach, four lncRNA expression-based subtypes were discovered in the Clinseq-AML cohort and validated in an independent (TCGA-AML) cohort. The distribution of molecular and clinical data by the lncRNA-based consensus clusters is shown in Fig. 1.

**Table 1:** Description of Clinseq-AML cohort

Number of patients	274
Sex: no. of patients (%)	
Male	133 (48.5%)
Female	141 (51.4%)
Age	
Median (range)	64.5 (18–85)
No. of the patients aged < 60	109 (39.7%)
Etiology	
De novo AML	222
s-AML	24
t-AML	26
Missing	2
Median follow-up (days)	346.5
Bone marrow blast: median (range, %)	53.5% (14–100%)
WBC counts: median (range, per mm^3^)	20.5 (0.5–298.4)
ELN	
High	53
Intermediate	142
Low	73
Cytogenetic aberrations: *N* (%)	
t(15;17)	8 (2.9%)
t(8;21)	5 (1.8%)
inv(16)/t(16;16)	9 (3.3%)
Normal	130 (47.4%)
inv(3)/t(3;3)	5 (1.8%)
Complex	32 (11.7%)
del(5)	17 (6.2%)
del(7)	27 (9.9%)
t(11q23)	7 (2.6%)
Mutation: *N* (%)	
ASXL1	28 (10.22%)
CEBPA	39 (14.23%)
CEBPA (double)	17 (6.2%)
DNMT3A	63 (22.99%)
FLT3-TKD	77 (28.1%)
FLT3-ITD	68 (24.82%)
IDH1	28 (10.22%)
IDH2	50 (18.25%)
KRAS	11 (4.01%)
NPM1	83 (30.29%)
RUNX1	39 (14.23%)
TET2	62 (22.63%)
TP53	24 (8.76%)
WT1	9 (3.28%)

*Abbreviations*: *WBC* white blood cell, *ELN* European LeukemiaNet, *FLT3-ITD* internal tandem duplication of the FLT3 gene, *FLT3-TKD* tyrosine kinase domain mutation in the FLT3 gene, *t-AML* therapy-related acute myeloid leukemia, *s-AML* secondary acute myeloid leukemia

### Individual lncRNAs are prognostic of overall survival in AML

**Fig. 2:**
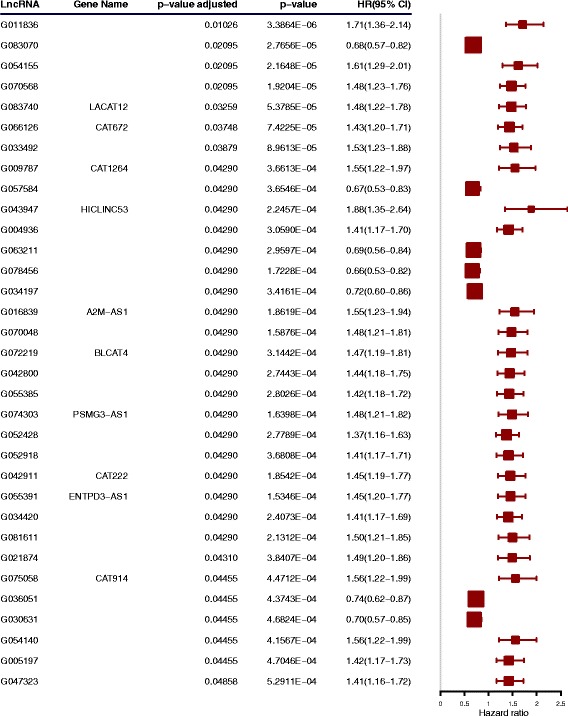
Multivariate time-to-event analysis (overall survival) of individual lncRNA (adjusting for established risk factors) in the Clinseq-AML cohort

First, we investigated to what extent individual lncRNAs were associated with overall survival in the Clinseq-AML cohort. Individual Cox proportional hazards regression models were fitted for each lncRNA using time-on-study as the time scale, adjusting for age, sex, ELN risk score, mutation status of CEBPA, NPM1, TP53, WT1, TET2, ASXL1, DNMT3A, RUNX1, IDH1, IDH2, and FLT3-ITD, and chromosomal abnormalities as covariates in the models. We found 33 prognostic (overall survival) lncRNAs (adjusted *p* value < 0.05, Fig. 2). These results suggest that there are individual lncRNAs that provide prognostic information beyond established risk classification scores (ELN risk score) and typical somatic aberrations in AML. We analyzed the association between lncRNA expression and overall survival in the TCGA-AML cohort (Additional file 1: Figure S1). However, none of the association have significant *p* value (< 0.05). A possible reason might be the small sample size of the TCGA-AML dataset.

### Novel lncRNA-based molecular subtypes of AML

**Fig. 3:**
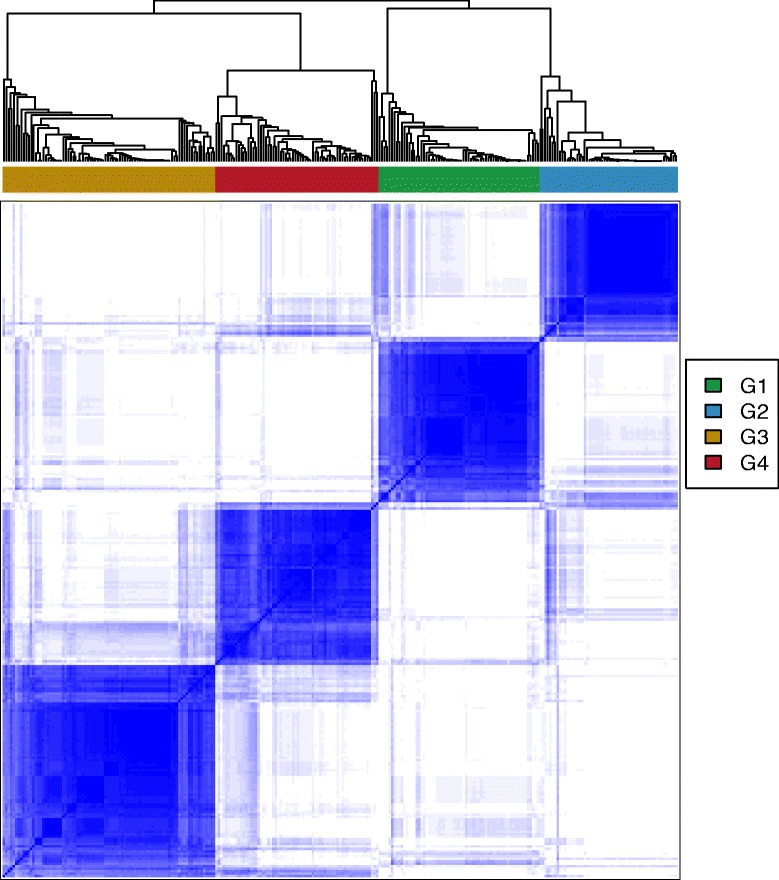
Consensus clustering matrix for 4 groups. Comparing different number of clusters indicates *K* = 4 is the optimal number of clusters in lncRNA expression dataset. (Model selection results for *K* = 2 to 8 is provided in Additional file 1: Figures S2–S4)

Next, we investigated if subgroups of AML patients were present in the Clinseq-AML cohort that shares common multivariate lncRNA expression patterns. We applied an unsupervised consensus clustering approach (see the “Methods” section) to the lncRNA expression profiles and discovered four distinct lncRNA-based subtypes. Consensus clustering results indicated a high degree of co-clustering of subjects within these four groups (Fig. 3 and Additional file 1: Figures S2–S4). This indicates that AML patients in the Clinseq cohort could be stratified into four distinct subtypes based on their lncRNA expression abundances.

### LncRNA AML subtypes are prognostic

**Fig. 4:**
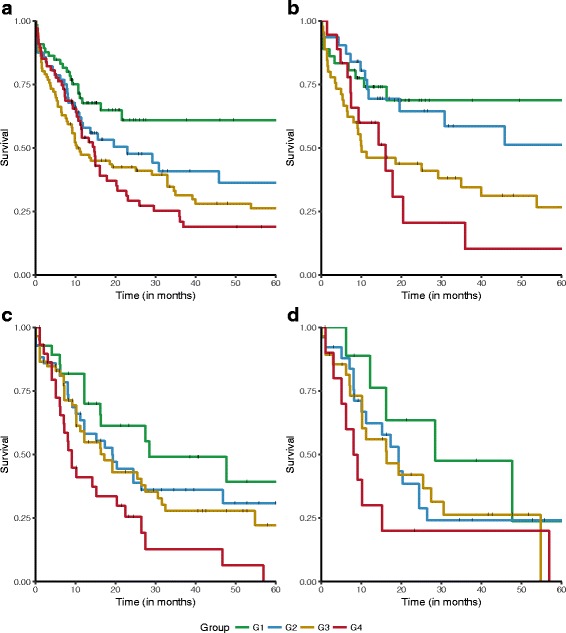
LncRNA expression subtypes and overall survival (OS). **a** OS (Kaplan-Meier) in the full Clinseq-AML cohort (*p* value = 0.04, log-rank test). **b** OS (Kaplan-Meier) in cytogenetically normal patients in the Clinseq-AML cohort (*p* value = 0.02, log-rank test). **c** OS (Kaplan-Meier) in the TCGA-AML cohort (*p* value = 0.01, log-rank test). **d** OS (Kaplan-Meier) in cytogenetically normal patients in the TCGA-AML cohort (*p* value = 0.2, log-rank test)

We assessed the prognostic information of the lncRNA-based subtypes in respect to overall survival (Fig. 4 and Additional file 1: Figure S5) and event-free survival (Additional file 1: Figure S6). The prognostic value of the four lncRNA-based subtypes was found to be significant (*n* = 274, *p* = 0.04 (log-rank test)). Among patients in cluster G1 (*N* = 65), the mean (±SE) overall survival at 60 months (5 years) was 61 ± 7%. Patients in clusters G2 and G3 had an intermediate rate of overall survival of 36 ± 8% and 26 ± 5% respectively. The cluster G4 has the worst survival outcome with an overall survival at 60 months of 18 ± 5%. The prognostic performance was also evaluated in the subset of cytogenetically normal patients (*N* = 130), which confirmed that the lncRNA-based subtypes were significantly associated with overall survival also in this subpopulation (Fig. 4b, *p* value = 0.02, log-rank test). Event-free survival for the lncRNA-based subtypes (Additional file 1: Figure S6) also provides a significant prognostic value (*p* = 0.015 (log-rank test)).

We validated the lncRNA expression-based subtypes in the independent TCGA-AML cohort (Fig. 4c, d). In the TCGA-AML cohort, the prognostic value of the lncRNA-based subtypes is significant (Fig. 4c, *n* = 172, *p* = 0.01, log-rank test). However, for cytogenetically normal patients in the TCGA-AML cohort, the prognostic performance is not significant (Fig. 4d, *p* value = 0.2, log rank) which potentially might have occurred due to the low sample size (*n* = 78). Details of validation using the TCGA-AML cohort are provided in the following section.

**Fig. 5:**
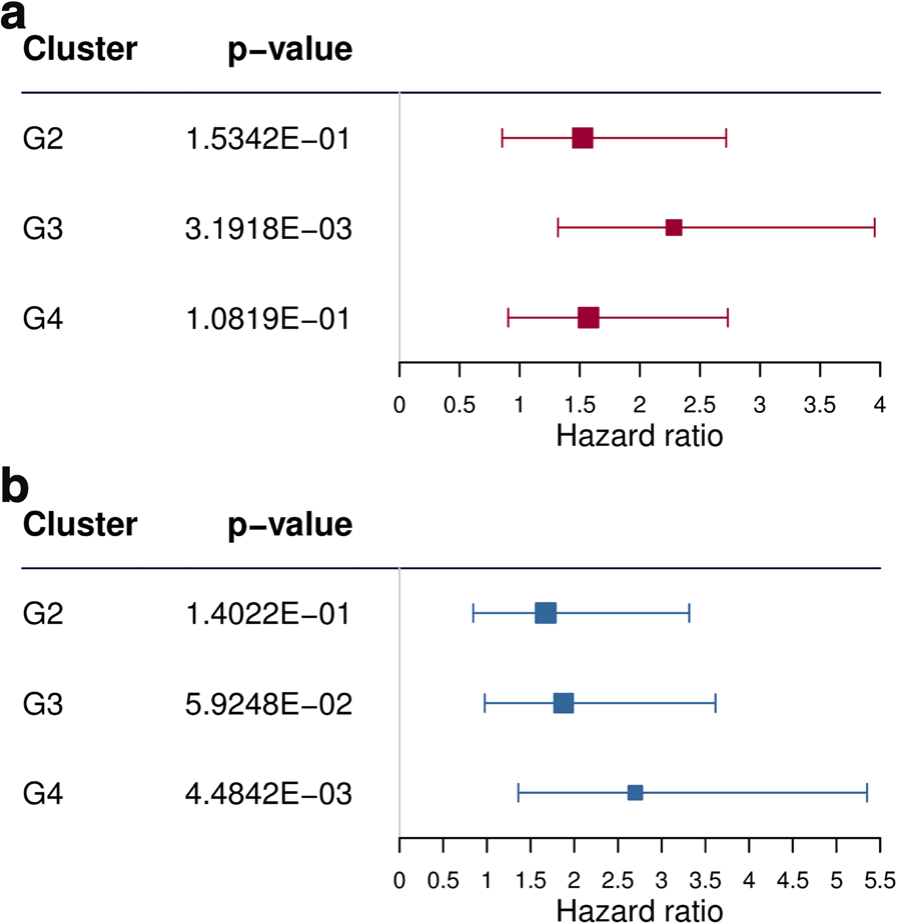
Multivariate survival analysis (Cox proportional hazards model) of lncRNA subtypes including age, sex, ELN risk score, mutation status of CEBPA, NPM1, TP53, WT1, TET2, ASXL1, DNMT3A, RUNX1, IDH1, IDH2, and FLT3 internal tandem duplications, and chromosomal abnormalities as covariates for **a** Clinseq-AML cohort and **b** TCGA-AML cohort

To ascertain if the subtypes were prognostic beyond established prognostic factors, we also fitted a multivariable Cox proportional hazards models, adjusting for established prognostic markers (Fig. 5), and this model was also found to provide a significant prognostic value (*p* value = 7.0 × 10^−7^). In particular, cluster G3 in this model was significantly different in overall survival compared with the reference group G1 (*p* value = 3.2 × 10^−3^, Fig. 5a).

### Nested cross-validation and independent validation of the lncRNA subtype

To determine consistency of the subtype discovery, we implemented a nested cross-validation procedure that is analogous to repeatedly splitting our cohort into a training set (for model fitting, including parameter estimation) and an independent subset of patients for model evaluation in respect to prognostic value (test set). The misclassification rate of test set samples (nested cross-validation) was low, with overall classification accuracy in the nested cross-validation procedure of 85% (Additional file 1: Figure S7), using class labels assigned in the primary subtype discovery phase as reference. Cross-validation of lncRNA subtypes also revealed significant prognostic value (overall survival, Additional file 1: Figure S8) (*p* value = 0.012). These results indicate that the lncRNA subtypes, prediction model, and the prognostic value of the subtypes are robust.

Next, we assessed the reproducibility of newly discovered AML subtypes using independent TCGA-AML cohort. To handle intrinsic batch differences between the Clinseq and TCGA studies, we applied batch correction on Clinseq and TCGA lncRNA expression data [25]. We trained a random forest model [26] for subtype classification based on the Clinseq data and subsequently predicted subtypes in the TCGA-AML cohort. A list of lncRNAs selected using random forest models can be found in Additional file 2. Based on the predicted subtype labels in the TCGA cohort, we then assessed the prognostic information in respect to overall survival (Fig. 4c and Additional file 1: Figure S5B). In the TCGA-AML cohort, the prognostic value of the four lncRNA-based subtypes was found to be significant (*n* = 172, *p* = 0.01, log-rank test). In concordance with Clinseq-AML cohort, in TCGA-AML cohort, subtype G1 (*n* = 30) has the best survival outcome with mean (SE) overall survival at 60 months (5 years) which is 40 ± 12%. Similarly, subtypes G2 (*n* = 43) and G3 (*n* = 69) show intermediate survival with mean (SE) overall survival at 60 months which are 31 ± 8% and 22 ± 87% respectively. Similar to Cliniseq, subtype G4 (*n* = 30) has the worst survival outcome in TCGA cohort, where no patient survive at 60 months (Fig. 4c). We also fitted a multivariable Cox proportional hazards models, adjusting for age, sex, and established prognostic markers using TCGA clinical and mutation data (Fig. 5b). Prognostic value of this model was also found to be significant value (*p* value = 1.25 × 10^−9^). When compared with the reference group G1, in this model, subtype G4 was significantly different in overall survival (*p* value = 4.48 × 10^−3^). We also evaluated the prognostic performance in the subset of cytogenetically normal patients in the TCGA-AML cohort. In this subset of patients, the association to overall survival was not significant (Fig. 4d, *p* value = 0.2, log rank). However, this might be due to the low sample size (*n* = 78).

### LncRNA expression subtypes are partially associated with clinicopathological factors

To determine if the lncRNA-based subtypes were associated with known cytogenetic or mutational aberrations, we applied association tests between subtypes and key genetic aberrations and clinical phenotypes (Table 2). Neither of the subtypes was found to be highly concordant with any of the conventional clinical or genetic factors (for details, see Additional file 1: Tables S1 to S20).

**Table 2:** Association analysis of lncRNA-derived molecular subtypes with established somatic aberrations and other risk factors

Cluster	G1	G2	G3	G4	*p* value	Adjusted *p* value
Patients	65 (23.72%)	56 (20.44%)	86 (31.39%)	67 (24.45%)		
Mutation						
FLT3_ITD	15(5.47%)	12(4.38%)	37(13.50%)	4(1.46%)	2.88E−06	3.60E−05
NPM1	18(6.57%)	19(6.93%)	40(14.60%)	6(2.19%)	1.09E−05	9.08E−05
TP53	5(1.82%)	3(1.09%)	3(1.09%)	13(4.75%)	3.95E−03	1.98E−02
KRAS	3(1.09%)	5(1.82%)	0(0.00%)	3(1.09%)	5.81E−02	1.45E−01
IDH1	12(4.38%)	2(0.73%)	8(2.92%)	6(2.19%)	5.25E−02	1.45E−01
RUNX1	14(5.11%)	7(2.56%)	7(2.56%)	11(4.01%)	1.19E−01	2.70E−01
WT1	1(0.36%)	1(0.36%)	6(2.19%)	1(0.36%)	1.36E−01	2.83E−01
FLT3-TKD	15(5.47%)	15(5.47%)	41(14.96%)	6(2.19%)	1.92E−06	3.60E−05
ASXL1	6(2.19%)	8(2.92%)	5(1.82%)	9(3.29%)	3.02E−01	4.19E−01
CEBPA	11(4.01%)	6(2.19%)	15(5.47%)	7(2.56%)	4.82E−01	5.48E−01
CEBPA (double)	4(1.46%)	1(0.36%)	8(2.92%)	4(1.46%)	3.63E−01	4.78E−01
IDH2	16(5.84%)	9(3.29%)	16(5.84%)	9(3.29%)	3.93E−01	4.91E−01
TET2	14(5.11%)	16(5.84%)	21(7.66%)	11(4.01%)	4.22E−01	5.02E−01
DNMT3A	16(5.84%)	14(5.11%)	17(6.20%)	16(5.84%)	8.59E−01	8.67E−01
Cytogenetic aberrations						
Normal karyotype	36(13.79%)	31(11.88%)	45(17.24%)	18(6.90%)	3.96E−03	1.98E−02
inv(16)/t(16;16)	0(0.00%)	2(0.77%)	1(0.38%)	6(2.30%)	9.05E−03	3.23E−02
del5	5(1.92%)	2(0.77%)	1(0.38%)	9(3.45%)	7.85E−03	3.23E−02
del7	7(2.68%)	1(0.38%)	7(2.68%)	12(4.60%)	1.53E−02	4.78E−02
t(8;21)	0(0.00%)	1(0.38%)	1(0.38%)	3(1.15%)	2.32E−01	3.75E−01
+ 8	2(0.77%)	5(1.92%)	4(1.53%)	7(2.68%)	2.40E−01	3.75E−01
t(11q23)	0(0.00%)	3(1.15%)	2(0.77%)	2(0.77%)	2.94E−01	4.19E−01
t(15;17)	3(1.15%)	1(0.38%)	4(1.53%)	1(0.38%)	6.60E−01	7.17E−01
inv(3)/t(3;3)	1(0.38%)	1(0.38%)	1(0.38%)	2(0.77%)	8.67E−01	8.67E−01
ELN risk						
High	16(6.15%)	10(3.85%)	16(6.15%)	21(8.08%)	2.11E−01	3.75E−01
Intermediate	37(14.23%)	32(12.31%)	53(20.39%)	27(10.38%)		
Low	9(3.46%)	13(5.00%)	14(5.38%)	12(4.62%)		
Etiology						
De novo	48(17.65%)	45(16.54%)	73(26.84%)	56(20.59%)	1.52E−01	2.92E−01
s-AML	4(1.47%)	4(1.47%)	8(2.94%)	8(2.94%)		
t-AML	11(4.04%)	7(2.57%)	5(1.84%)	3(1.10%)		

Percentages refer to entire cohort

Patients belonging to group G1 are enriched for CEBPA mutations (2.56 and 1.46% single and double mutation respectively). CEBPA double mutations have been associated with favorable outcome in AML [27, 28]. Cluster G2 is enriched in NPM1 mutation (6.93%) but has low percentage of TP53 mutations (1.09%). The cluster G3 contains a substantial number of FLT3-ITD. This cluster is also enriched in CEBPA single and double mutations. Cluster G4 harbors a high percentage of TP53 mutations (4.75%). This cluster also contains the highest percentage (8.08%) of patients classified as high-risk category using ELN risk classification system.

We found that lncRNA expression-based subtypes were independent from the European LeukemiaNet (ELN) risk classification system [4] and the distribution of the ELN risk score is fairly even in all four groups (Fig. 1 and Additional file 1: Table S19). For each ELN risk type, we further stratified it using lncRNA subtypes (Additional file 1: Figure S9). These results indicate that for each ELN risk score, lncRNA subtypes can provide further stratification of patients. Although lncRNA-based subtypes were not found to be highly concordant with any specific mutations, cytogenetics, or clinical factors, we found that mutations in NPM1 and TP53 were associated with the lncRNA-based subtypes (Chi-square test *p* value is 1.09 × 10^−5^ and 2.99 × 10^−3^ respectively, see Additional file 1: Tables S1 to S20 for details).

### Pathway analysis of genes associated with lncRNA-based subtypes

**Fig. 6:**
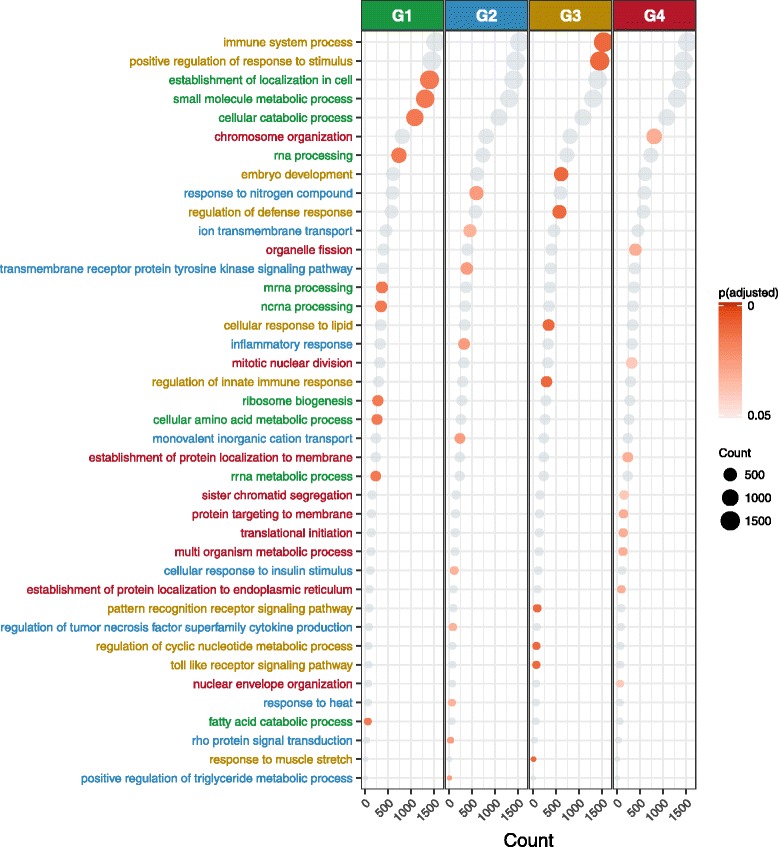
Top ten pathways uniquely enriched in each subtype. Count represents the number of genes found in each pathway, and *p*.adjust is the Benjamini and Hochberg FDR-corrected *p* value of the overrepresentation test. A list of all pathways, corresponding *p* values, and FDR-adjusted *p* value can be found in Additional file 3

LncRNAs have very limited functional assignments. In order to gain some overview of potential molecular mechanism related to the lncRNAs that define the lncRNA-based subtypes, we performed pathway analysis. First, we determined which mRNA transcripts were associated with the lncRNA-based subtypes, and subsequently, we utilized this set of mRNAs for pathway enrichment analysis (see the “Methods” section for details). This analysis revealed multiple significant pathways (Fig. 6) include “immune system” (adjusted *p* value = 0.01), “chromosome organization” (adjusted *p* value = 0.03), “mRNA processing” (adjusted *p* value = 0.01), and “transmembrane receptor protein tyrosine kinase signaling pathway” (adjusted *p* value = 0.02). List of all pathways and differentially expressed genes in four clusters can be found in Additional files 3 and 4 respectively.

### LncRNA-based subtypes are not concordant with mRNA-based subtypes

**Fig. 7:**
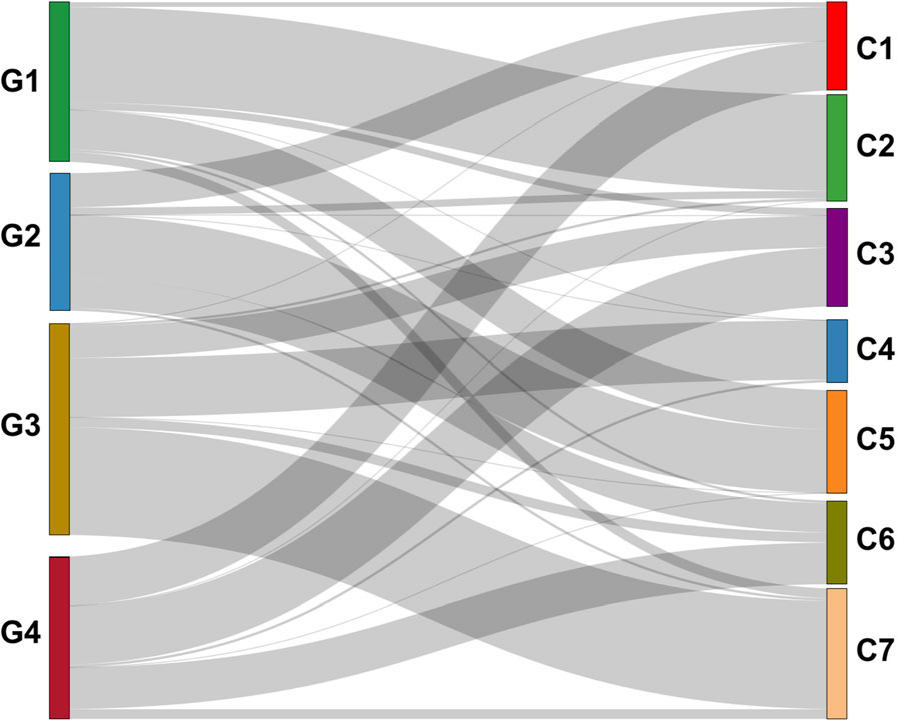
Sankey diagram of the relationship between mRNA-defined subtype classification (right) and lncRNA-defined subtypes (left) in AML. Each block on the left side represents the lncRNA subtypes with bar height proportional to the number of patients in each group. Each block on the right side represents mRNA subtypes, and lines connecting right and left side indicate the relationship between lncRNA and mRNA subtypes

Since lncRNA expression levels can in some cases be correlated to the expression levels of cis-located mRNAs [29] and potentially also be correlated with the global mRNA expression profile, we evaluated to what extent lncRNA-based subtypes were reflected in mRNA-based expression clusters. We applied an identical unsupervised consensus clustering methodology to determine mRNA-based clusters as for the lncRNA analysis (see Additional file 5 supplementary methods for details). Despite stratifying patients into groups that are substantially different (Fig. 7), a Chi-square test of dependence between mRNA and lncRNA subtype models did allow us to reject the null hypothesis of no relationship between the models (*p* value = 2.56 × 10^−68^; Additional file 1: Table S21). For instance, mRNA subtype C2 is almost fully subsumed in lncRNA subtype G1, which might be a substantial contributor to the Chi-square statistic in this case. However, despite that mRNA and lncRNA models cannot be considered as independent, we note that mRNA and lncRNA expression profiling data stratify patients into markedly different groups (Fig. 7), suggesting that the information in mRNA and lncRNA expression profiles are different.

## Discussion

The present study is the most comprehensive lncRNA expression study in AML to date. We characterized lncRNA expression using RNA sequencing in a cohort of 274 AML patients (data included in Additional file 6) with the aim to determine if individual lncRNAs were associated with AML outcome and if lncRNA-based prognostic subtypes of AML could be defined. The findings were subsequently validated in the independent TCGA-AML cohort (Additional file 7).

In the Clinseq-AML cohort, 33 individual lncRNAs were found to have independent prognostic information and four robust lncRNA-based subtypes of AML were discovered that are prognostic of overall survival. Some of the established clinical and genetic factors of AML were found to be associated with the lncRNA expression subtypes, although subtypes did not display a high degree of concordance with any of the clinical or genetic factors. Similarly, lncRNA-based subtypes were not found to be concordant with mRNA-based subtypes, suggesting that lncRNA expression represents an independent source of molecular information. Subtype G1 was characterized by displaying the longest overall survival. This group is also dominated by intermediate level of ELN risk and normal karyotypes. It also harbors high frequency of CEBPA double mutations. In de novo AML, CEBPA double mutations are known to have a favorable prognostic significance [27, 28]. Subtypes G2 and G3 represent prognostically poorer AML subtypes. Both of these subtypes have a high frequency of patients with intermediate risk level based on ELN risk classification. In comparison to subtype G1, they possess more cytogenetic abnormalities. Subtype G4 represents a group of AML patients with poor prognosis, with the highest frequency of TP53 single and double mutations. When ascertaining the independent prognostic value of lncRNA subtypes, given ELN risk classification (which includes cytogenetic classification), and genetic mutations, the lncRNA subtype model was confirmed to provide a significant prognostic value. We have also developed a subtype prediction biomarker panel consisting of 35 lncRNAs (Additional file 2), which provided equivalent classification as the full set of lncRNA features considered in this study and could be seen as a candidate biomarker panel for lncRNA-based subtyping in AML.

We have validated our lncRNA expression-based subtype model in independent TCGA-AML cohort. Our results show that similar to Clinseq-AML cohort, in the TCGA-AML cohort, the lncRNA-based subtypes are significantly associated with overall survival. In particular, it is evident that subtype G1 is associated with more favorable outcome and subtype G4 indicates worse outcome. These associations are evident in both the cohort even after adjusting for known prognostic factors through multivariate analysis.

Both Clinseq-AML and TCGA-AML cohorts have similar percentage of cytogenetically normal patients, 47.4 and 45.1% respectively. Cytogenetic abnormalities, such as del7 (9.9% in Clinseq-AML, 9.9% in TCGA-AML) and del5 (6.2% in Clinseq-AML, 5.6% in TCGA-AML), have very similar distribution in both the cohorts. However, frequency of recurrent genetic abnormalities such as inv(16) (3.3% in Clinseq-AML, 7.7% in TCGA-AML) and inv(3) (1.8% in Clinseq-AML, 0% in TCGA-AML) are not similar. Interestingly, the Clinseq-AML cohort contains both de novo and non-de novo AML patients; however, the TCGA-AML cohort is completely comprised of de novo AML cases. We performed differential gene expression analysis between de novo and non-de novo samples in the Clinseq-AML cohort (Additional file 8). However, we did not find any significant difference in lncRNA expression pattern between de novo and non-de novo AML as no lncRNA is significantly differentially expressed (fdr < 0.05).

We would like to stress the fact that there are several differences between the Clinseq and TCGA cohort such as difference is sequencing protocol, batch effect, and frequency of recurrent genetic abnormalities, as discussed above. Our analysis shows that despite the various sources of heterogeneity and cohort differences, lncRNA expression-based subtypes are consistent and have significant association with survival. Previously, Garzon et al. [7] studied lncRNA expression in cytogenetically normal acute myeloid leukemia (CN-AML) patients using a custom microarray platform with a focus on assessing lncRNAs association with routine clinical phenotypes and mutations. In contrast, present study contains a more representative set of AML patients and ascertains the presence of lncRNA-based molecular subtypes in AML. Furthermore, the present study is almost twice in compared to the previously published results [7], which only include CN-AML patients. We also note that RNA sequencing, which is employed here, provide an unbiased and comprehensive approach to lncRNA profiling compared to targeted microarray-based expression profiling which may be limited by selection bias during design of the array. Despite such differences, similar to Garzon et al. [7], our results show that pathways such as mRNA processing, immune system process, and chromosome organization are enriched in lncRNA subtypes G1, G3, and G4 respectively (Fig. 6 and Additional file 3).

We have also compared lncRNA expression-based subtypes with mRNA expression-based subtypes (C1 to C7). The mRNA subtypes were generated using the same methodology as lncRNA expression-based subtypes (for details, see Additional file 5). Our analysis shows that lncRNA-based subtypes are not directly correlated with mRNA-based subtypes and lncRNA subtypes provide independent prognostic information.

Although the present study is the largest lncRNA expression study reported to date, the sample size in this study might represent a limiting factor to establishing potential additional lncRNA subtypes that are rare (i.e., present in a low proportion of AML patients), since there would be too few principal examples present in this cohort. Furthermore, the RNAseq-based lncRNA profiling method applied in this study has limitations in quantifying lncRNA molecules at very low abundances. These limitations can be overcome by using a larger sample size and deeper sequencing technology.

## Conclusions

Expression profiles of lncRNAs have previously been studied in several cancer types, including proposed lncRNA subtypes [30–33]. However, in the context of hematological malignancies, only a few studies have focused on the role of lncRNA expression. Moreover, these studies have focused on risk prediction and were limited to a specific subset of AML. Our analysis is the first to provide lncRNA-based stratification of AML patients by means of lncRNA subtypes. The proposed subtypes are characterized by distinct molecular profiles defined by lncRNA expression, which also provide prognostic information. LncRNA expression and related molecular subtypes provide a promising avenue for improved patient stratification in the future and information about lncRNA expression that offer a starting point for functional studies.

## Methods

For detailed material and method, refer to the supplementary information provided in Additional file 5. A brief description is as follows:

### Patient cohorts

We used Clinseq-AML cohort, consist of 274 AML patients, treated according to the national guidelines in Sweden. The study was approved by the regional ethical review board in Stockholm, Sweden. All samples from the Clinseq-AML cohort were collected prior to the initiation of treatment. For detail characteristics of patients in Clinseq-AML cohort, see Table 1. In this study, we used data from 142 patients of the TCGA-AML study [1], who have received intensive induction treatment (chemotherapy) analogous to the Clinseq-AML cohort. Clinical and mutational data was retrieved from the data portal of TCGA (https://gdc.cancer.gov) and TCGA-AML study publication [1]. Detailed characteristics of TCGA-AML cohort can be found in Additional file 7.

### Sequencing and bioinformatics processing

Transcriptomic RNA and somatic mutation panel of genes were sequenced using the Illumina HiSeq-2500 platform. Ribosomal RNA depletion was performed using the Ribo-Zero gold kit. HTSeq count version 0.6.1 [34] was used for gene expression estimation. RNAseq count data normalization was performed using the TMM method [35]. A total of 3030 lncRNAs were annotated using MiTranscriptome database [6].

### Subtype discovery and validation

Consensus clustering-based unsupervised learning was applied for subtype discovery [24]. Optimal number of cluster (*k* = 4) was determined using weighted silhouette index. For validation, first, we performed 10-fold cross-validation on Cliniseq-AML data. At each cross-validation round, data was randomly divided into train and test set. Unsupervised learning was performed on training set, and labels were used to train random forest model [26]. Labels for test dataset were predicted using this model.

For independent validation, common lncRNA in Clinseq and TCGA dataset were selected as features and batch correction was applied [25]. We trained random forest classifier [26] on batch-corrected Clinseq-AML data and subtype labels were predicted for TCGA-AML data.

### Clinical association and survival analysis

For association analyses, Chi-square test was used. Overall survival was measured from the date of diagnosis to the date of death. Kaplan-Meier curve and non-parametric log-rank statistic were used for comparison. Uni-variable and multivariable Cox’s proportional hazards regression models were fitted to the survival data. In multivariate analysis, we adjusted for age, sex, etiology, ELN score, and mutational status of genes. Analysis was carried out using R (version 3.1.1).

## Additional files

Additional file 1: Supplementary figures and tables. Description of data: Figures and tables. (PDF 2723 kb)

Additional file 2: List of lncRNAs. Description of data: A list of lncRNAs selected using random-forest method. (XLSX 5 kb)

Additional file 3: List of pathways. Description of data: A list of pathways, corresponding *p*-values and FDR adjusted p-values. (XLSX 2434 kb)

Additional file 4: List of genes. Description of data: A list of differentially expressed genes in lncRNA subtypes. (XLSX 2264 kb)

Additional file 5: Supplementary methods. Description of data: Detailed description of the methods used in the analysis. (DOCX 66 kb)

Additional file 6: LncRNA expression data. Description of data: LncRNA expression data and corresponding AML subtypes. (CSV 8427 kb)

Additional file 7: Description of TCGA-AML cohort. Description of data: Clinical characteristics of the TCGA-AML cohort used in this study. (PDF 46 kb)

Additional file 8: lncRNA in de novo vs. non-de novo AML. Description of data: Differentially expressed lncRNA in de novo vs. non-de novo AML patients. (XLSX 13 kb)
