# Supplementary material for: Expression levels of long non-coding RNAs are prognostic for AML outcome
Source: J Hematol Oncol. 2018 Apr 7;11:52. doi: 10.1186/s13045-018-0596-2 (PMC5889529; doi:10.1186/s13045-018-0596-2)
Supplement: Supplementary file 1 — Supplementary figures and tables. Description of data: Figures and tables. (PDF 2723 kb) [file 13045_2018_596_MOESM1_ESM.pdf]

LncRNA	Gene Name	Clinseq p−value	Clinseq p−value(adj.)	Clinseq HR(95% CI)	TCGA p−value	TCGA p−value(adj.)	TCGA HR(95% CI)
G011836		3.3864E−06	0.01026	1.71(1.36−2.14)	1.7589E−01	0.39598	1.05(0.98−1.14)
G083070		2.7656E−05	0.02095	0.68(0.57−0.82)	2.5987E−01	0.50537	0.94(0.83−1.05)
G054155		2.1648E−05	0.02095	1.61(1.29−2.01)			
G070568		1.9204E−05	0.02095	1.48(1.23−1.76)			
G083740	LACAT12	5.3785E−05	0.03259	1.48(1.22−1.78)	3.6139E−01	0.60564	1.17(0.83−1.66)
G066126	CAT672	7.4225E−05	0.03748	1.43(1.20−1.71)			
G033492		8.9613E−05	0.03879	1.53(1.23−1.88)	2.2062E−01	0.46097	1.28(0.86−1.89)
G009787	CAT1264	3.6613E−04	0.04290	1.55(1.22−1.97)	4.4609E−01	0.67847	1.02(0.96−1.09)
G057584		3.6546E−04	0.04290	0.67(0.53−0.83)	9.0933E−03	0.09080	0.91(0.85−0.98)
G043947	HICLINC53	2.2457E−04	0.04290	1.88(1.35−2.64)	5.5962E−01	0.76408	1.02(0.95−1.10)
G004936		3.0590E−04	0.04290	1.41(1.17−1.70)	2.5718E−01	0.50209	1.04(0.97−1.12)
G063211		2.9597E−04	0.04290	0.69(0.56−0.84)	2.2844E−03	0.05108	0.88(0.81−0.96)
G078456		1.7228E−04	0.04290	0.66(0.53−0.82)	9.1911E−01	0.96523	1.00(0.91−1.09)
G034197		3.4161E−04	0.04290	0.72(0.60−0.86)	6.1390E−01	0.79965	0.96(0.84−1.11)
G016839	A2M−AS1	1.8619E−04	0.04290	1.55(1.23−1.94)	5.0582E−01	0.72054	0.96(0.84−1.09)
G070048		1.5876E−04	0.04290	1.48(1.21−1.81)	2.8736E−01	0.53259	0.93(0.81−1.06)
G072219	BLCAT4	3.1442E−04	0.04290	1.47(1.19−1.81)	4.3620E−01	0.67281	0.93(0.78−1.12)
G042800		2.7443E−04	0.04290	1.44(1.18−1.75)	6.2183E−01	0.80450	0.95(0.79−1.15)
G055385		2.8026E−04	0.04290	1.42(1.18−1.72)	2.3754E−01	0.48098	1.13(0.92−1.38)
G074303	PSMG3−AS1	1.6398E−04	0.04290	1.48(1.21−1.82)	4.7346E−01	0.69660	1.08(0.87−1.34)
G052428		2.7789E−04	0.04290	1.37(1.16−1.63)	3.3796E−01	0.58905	1.11(0.90−1.37)
G052918		3.6808E−04	0.04290	1.41(1.17−1.71)	5.9859E−01	0.79383	0.94(0.75−1.18)
G042911	CAT222	1.8542E−04	0.04290	1.45(1.19−1.77)	3.8836E−02	0.18842	0.73(0.55−0.98)
G055391	ENTPD3−AS1	1.5346E−04	0.04290	1.45(1.20−1.77)	2.8395E−01	0.53063	0.86(0.65−1.13)
G034420		2.4073E−04	0.04290	1.41(1.17−1.69)	9.7564E−01	0.99014	1.00(0.74−1.37)
G081611		2.1312E−04	0.04290	1.50(1.21−1.85)	9.7772E−01	0.99014	1.00(0.76−1.32)
G021874		3.8407E−04	0.04310	1.49(1.20−1.86)	4.3776E−01	0.67323	1.08(0.89−1.31)
G075058	CAT914	4.4712E−04	0.04455	1.56(1.22−1.99)	9.3378E−01	0.97221	1.00(0.92−1.10)
G036051		4.3743E−04	0.04455	0.74(0.62−0.87)	2.3815E−01	0.48183	0.93(0.82−1.05)
G030631		4.6824E−04	0.04455	0.70(0.57−0.85)	2.2072E−03	0.05065	0.87(0.79−0.95)
G054140		4.1567E−04	0.04455	1.56(1.22−1.99)	9.1180E−01	0.96214	1.01(0.88−1.16)
G005197		4.7046E−04	0.04455	1.42(1.17−1.73)	4.0772E−02	0.19253	0.75(0.57−0.99)
G047323		5.2911E−04	0.04858	1.41(1.16−1.72)	5.0772E−02	0.21238	0.69(0.48−1.00)

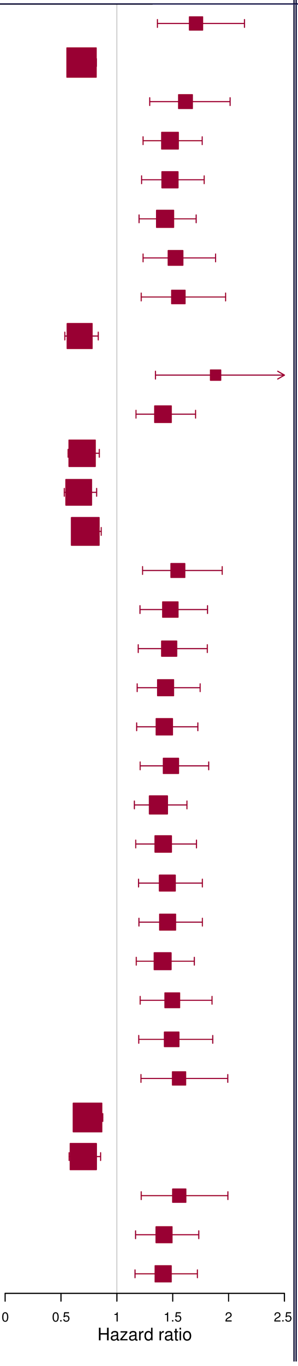

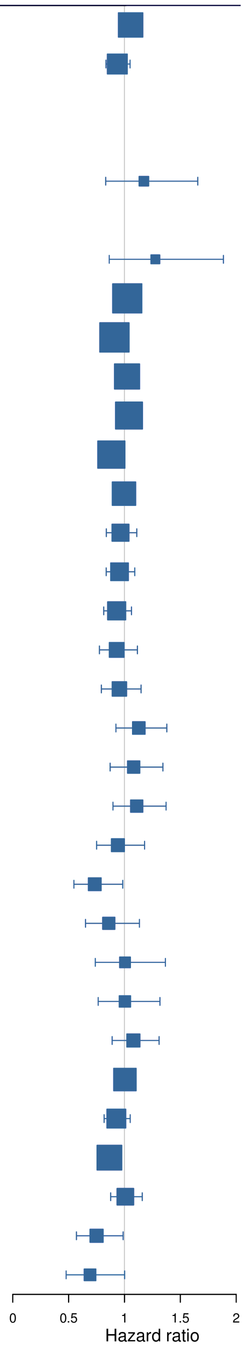

Figure S1: Multivariate time-to-event analysis (overall survival) of individual lncRNA (adjusting for established risk factors) in the Clinseq-AML (red) and TCGA-AML cohort (blue)

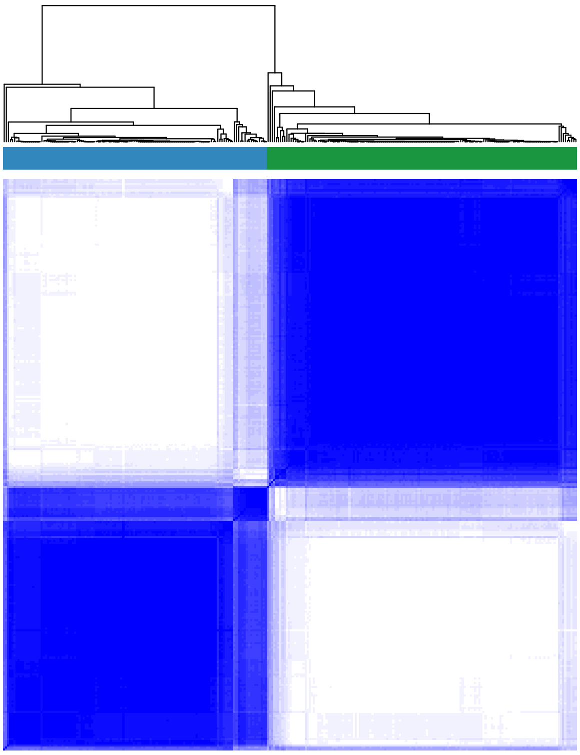

(a) K=2

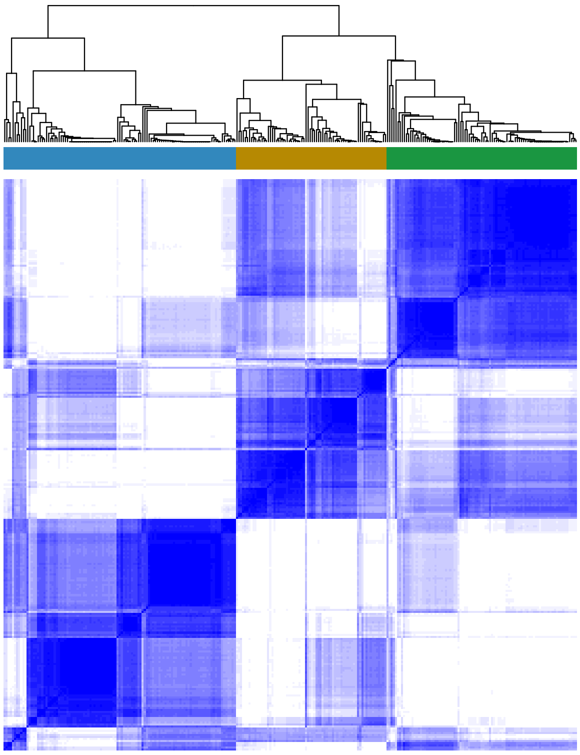

(b) K=3

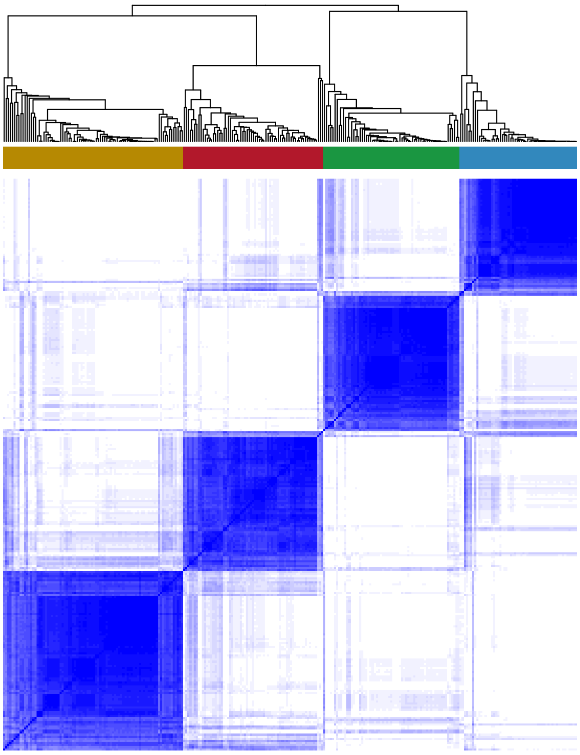

(c) K=4

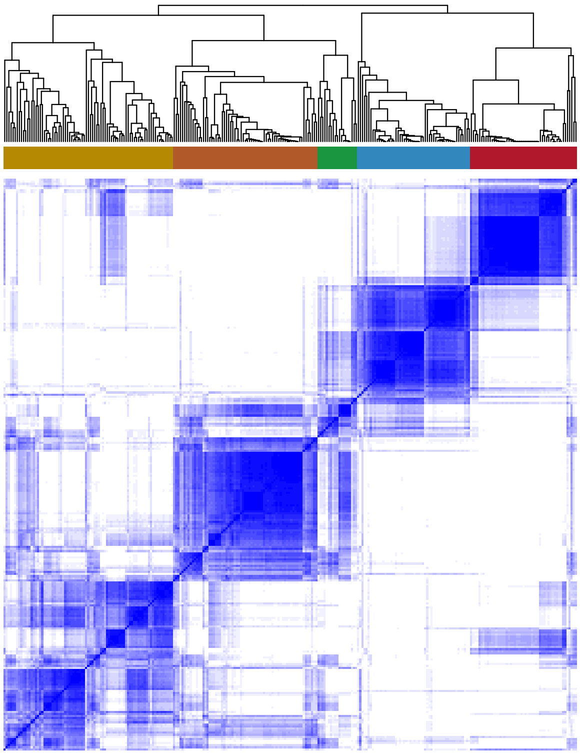

(d) K=5

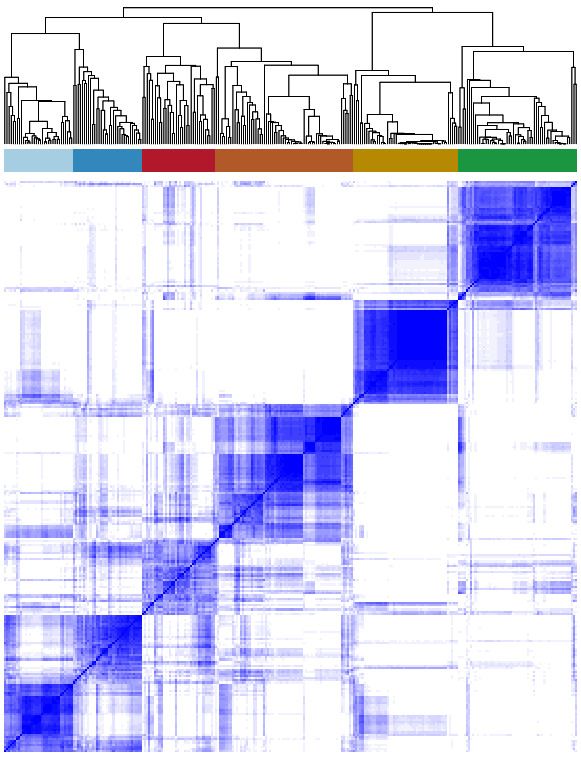

(e) K=6

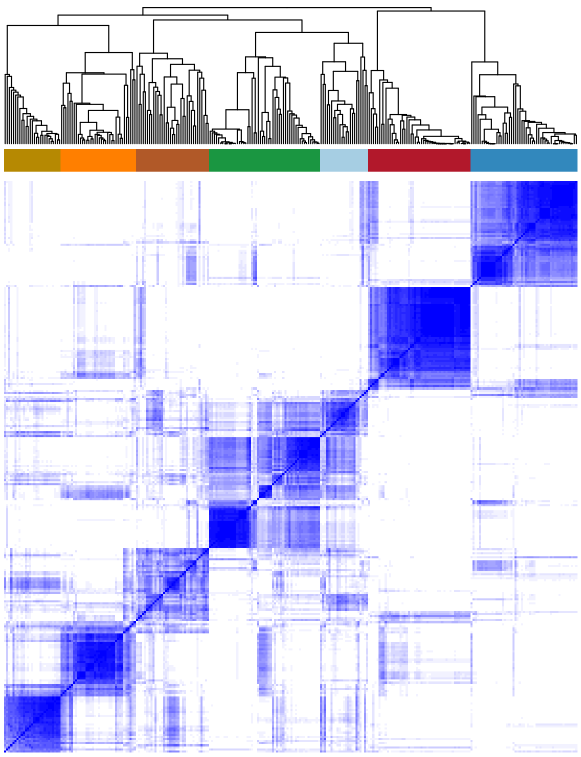

(f) K=7

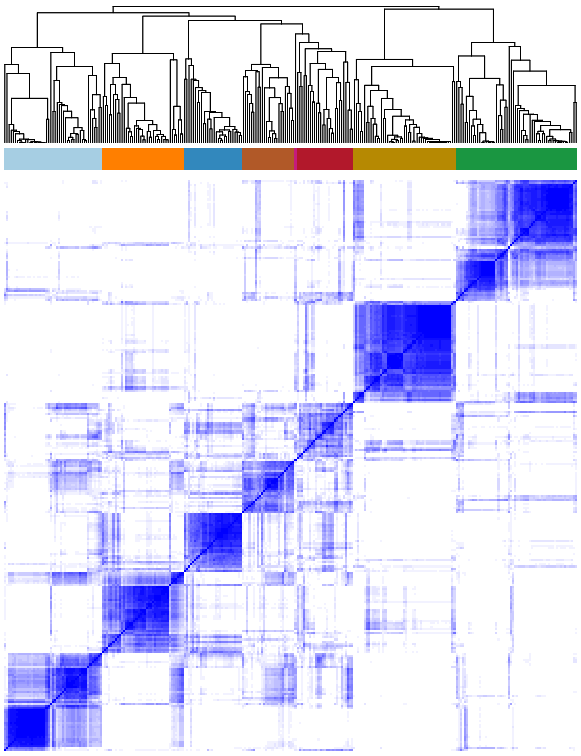

(g) K=8

Figure S2: Consensus matrix for clustering

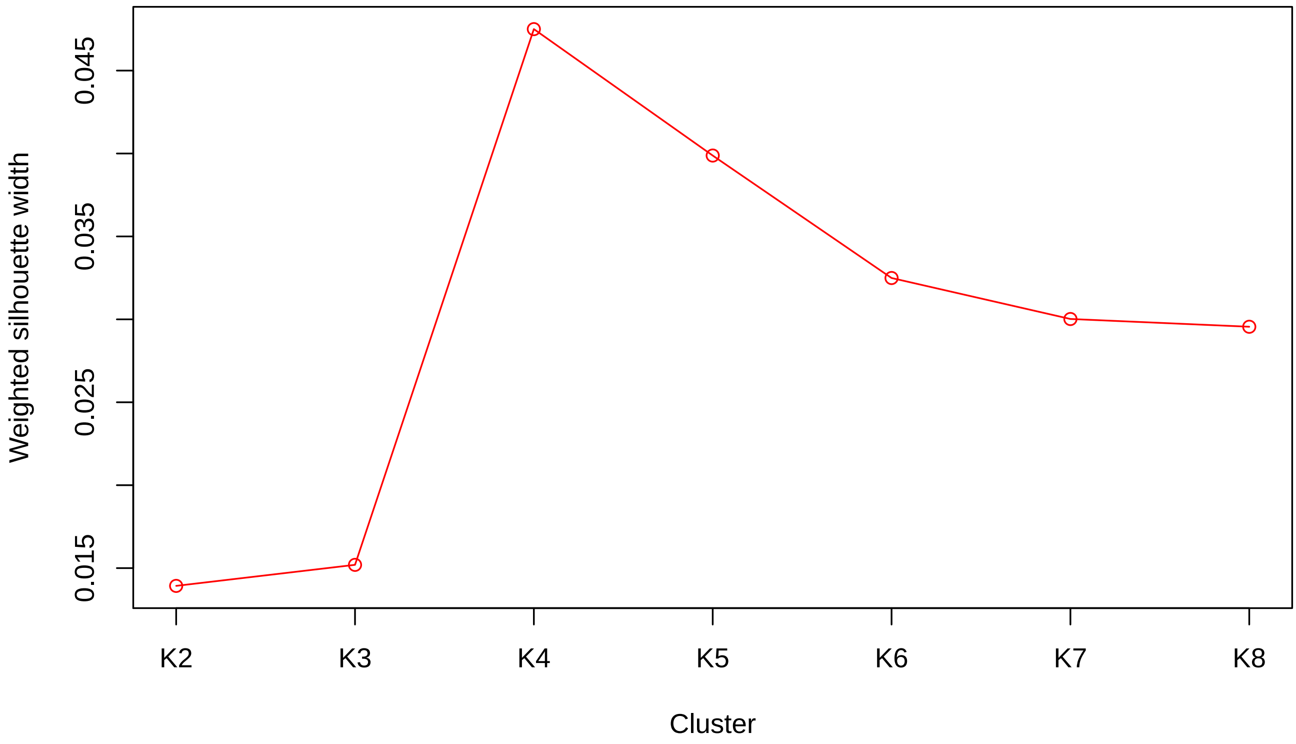

Figure S3: Weighted silhouette width for different number of clusters in lncRNA expression dataset

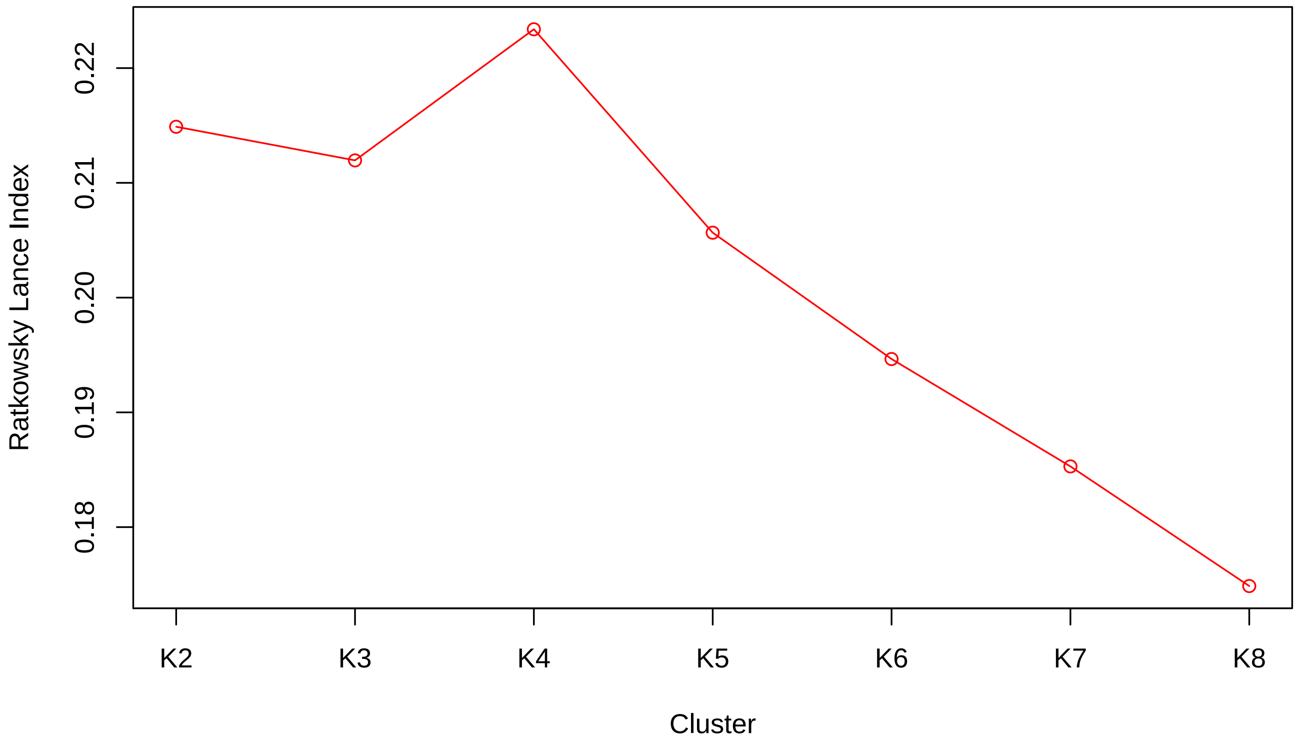

Figure S4: Clustering performance as a function of Ratkowsky Lance Index for different number of clusters in lncRNA expression dataset

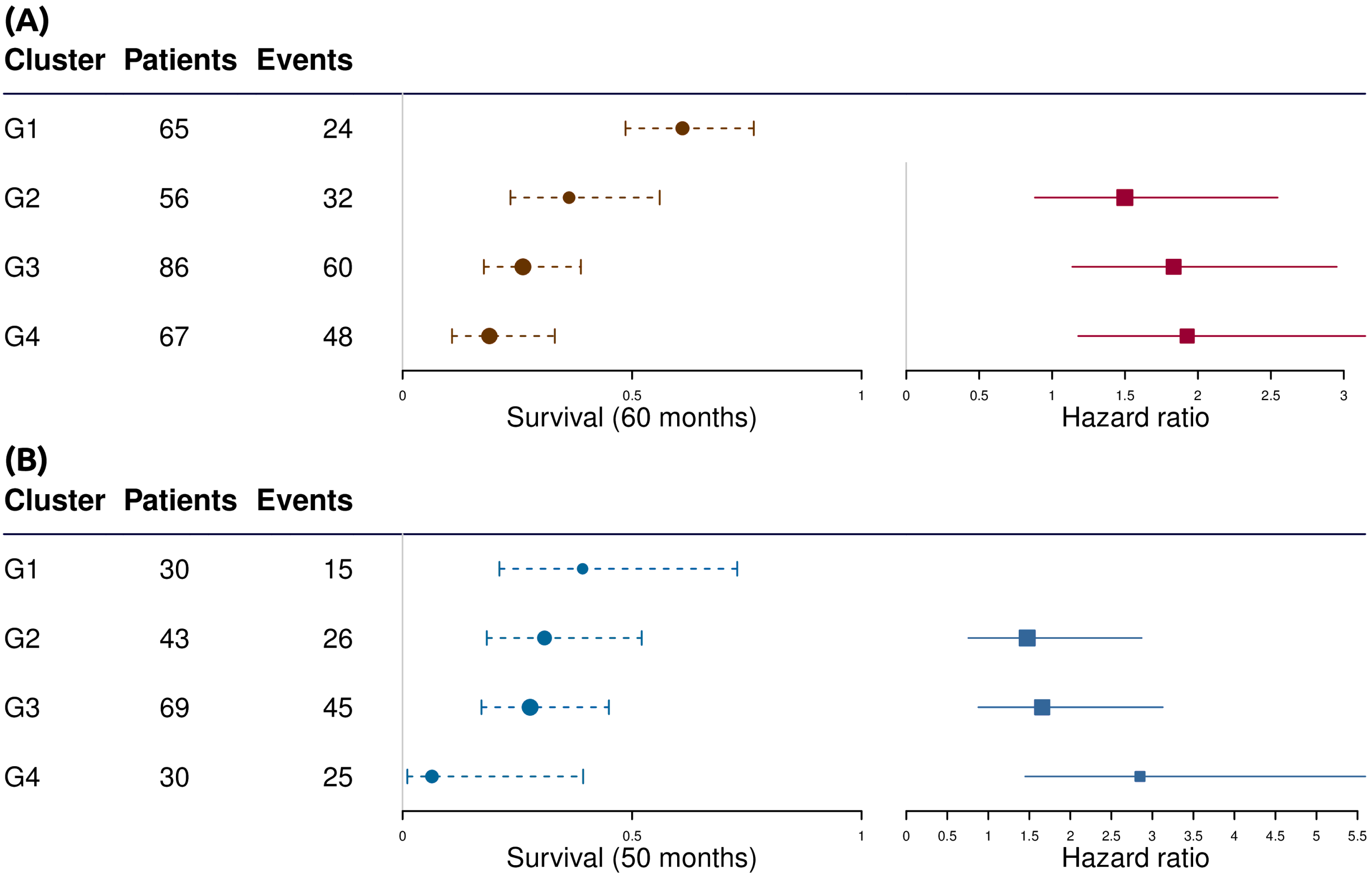

Figure S5: Hazard ratio and survival probability at 60 months in 4 different groups for (A) Clinseq AML cohort and (B) TCGA AML cohort. Barplot represent 95% confidence interval.

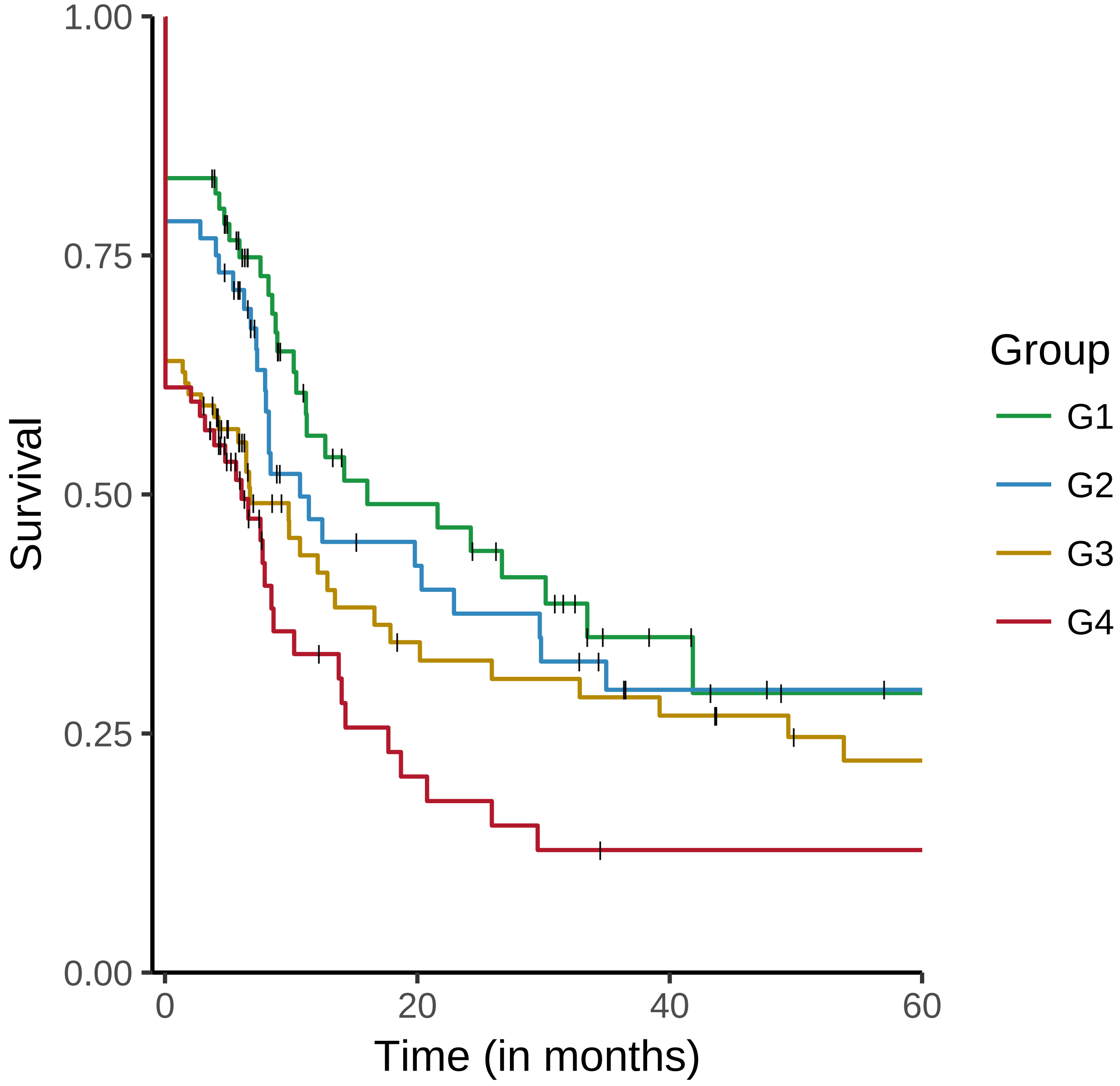

Figure S6: LncRNA expression subtypes and event free survival (Kaplan-Meier) in Clinseq AML cohort (p-value = 0.0159, Log-rank test).

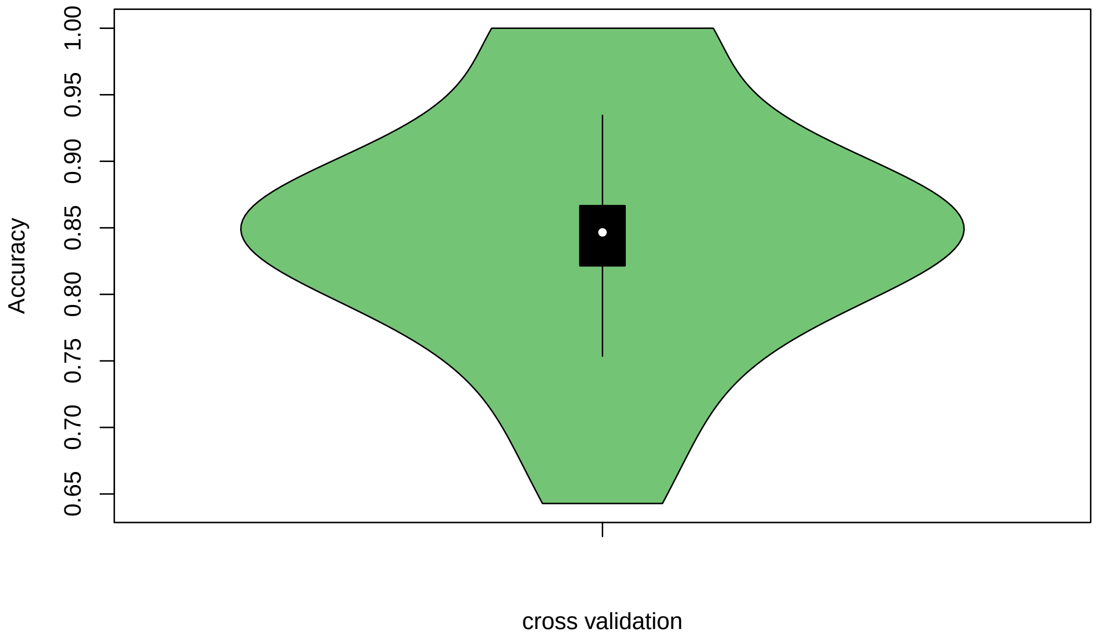

Figure S7: Violin plot visualisation for 100-fold cross-validation of lncRNA based AML subtype

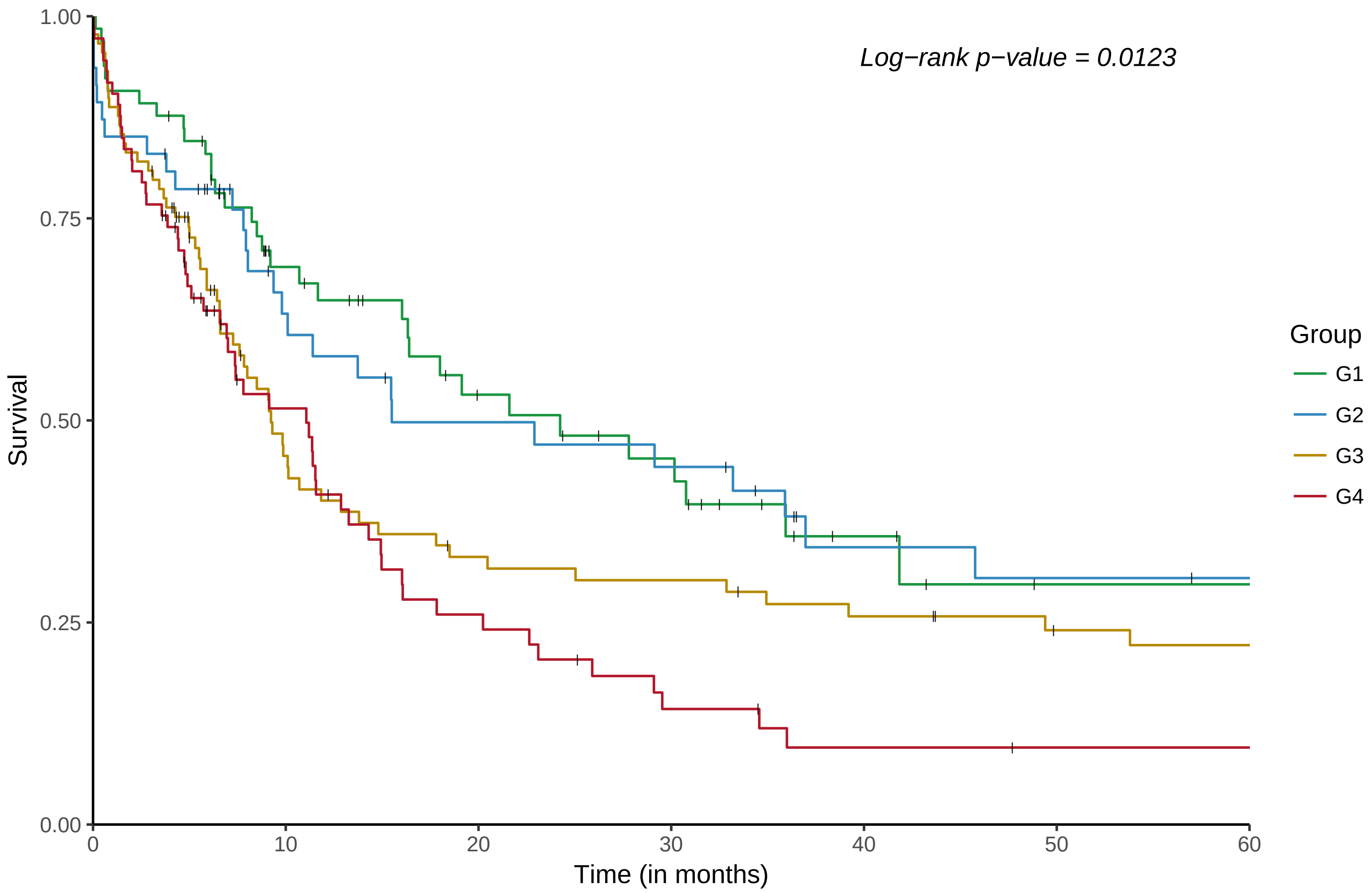

Figure S8: Overall survival (Kaplan-Meier) using predicted labels using 100-fold cross-validation in the complete Clinseq AML cohort

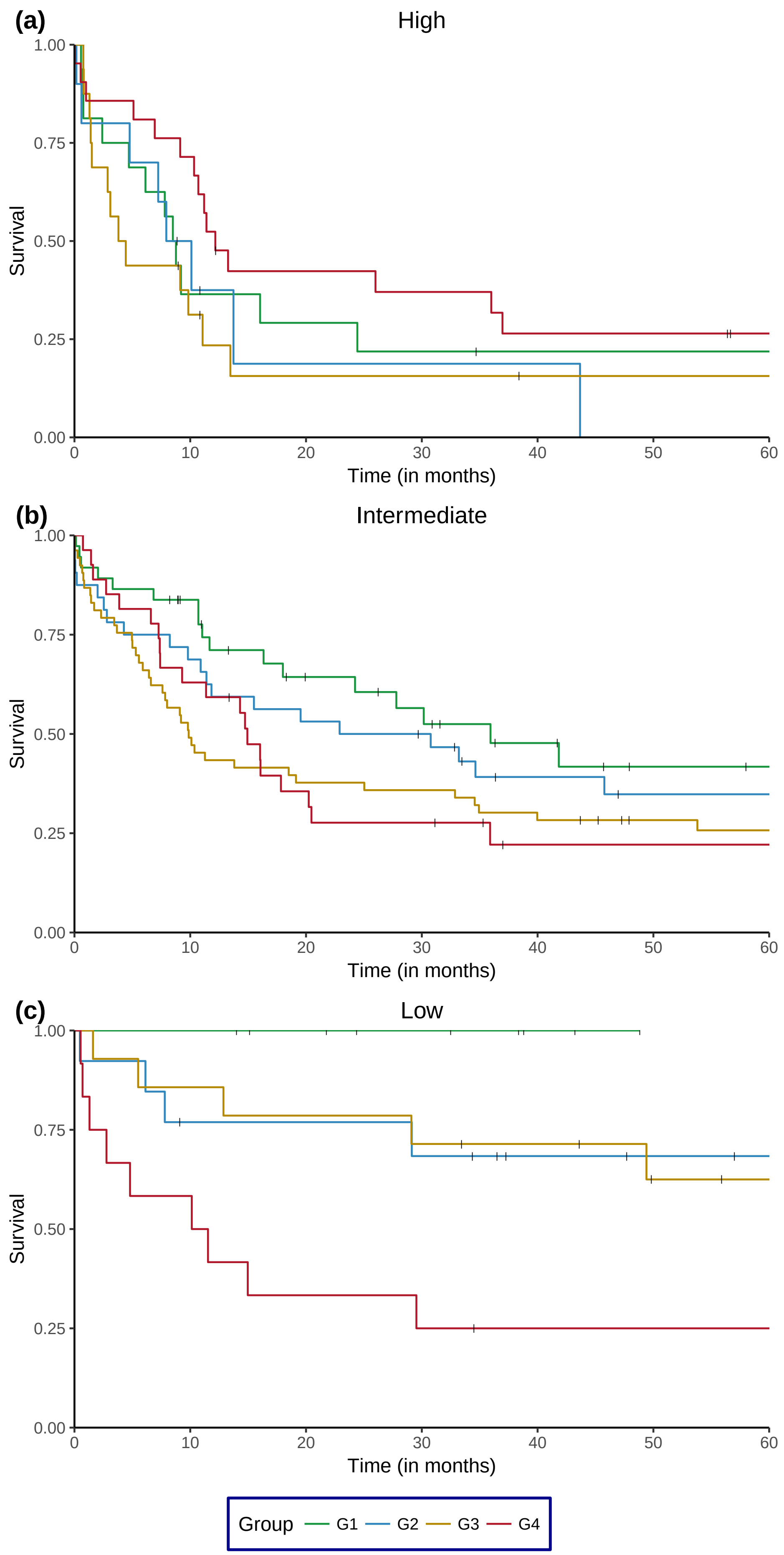

Figure S9: Overall survival (Kaplan-Meier) for 3 ELN risk groups, (a) high, (b) intermediate and (c) low further stratified by lncRNA based subtype

Table 1: CEBPA (p-value = 5.3242E-01)

	No mutation (in %)	Mutation (in %)	Double mutation (in %)
G1	19.71	2.56	1.46
G2	18.25	1.82	0.36
G3	25.91	2.56	2.92
G4	21.90	1.09	1.46

Table 2: NPM1 (p-value = 1.0924E-05)

	No mutation (in %)	Mutation (in %)
G1	17.15	6.57
G2	13.50	6.93
G3	16.79	14.60
G4	22.26	2.19

Table 3: TP53 (p-value = 3.9498E-03)

	No mutation (in %)	Mutation (in %)
G1	21.90	1.82
G2	19.34	1.09
G3	30.29	1.09
G4	19.71	4.75

Table 4: DNMT3A (p-value = 8.5920E-01)

	No mutation (in %)	Mutation (in %)
G1	17.88	5.84
G2	15.33	5.11
G3	25.18	6.20
G4	18.61	5.84

Table 5: KRAS (p-value = 4.9475E-02)

	No mutation (in %)	Mutation (in %)
G1	22.63	1.09
G2	18.61	1.82
G3	31.39	0.00
G4	23.36	1.09

Table 6: RUNX1 (p-value = 1.1877E-01)

	No mutation (in %)	Mutation (in %)
G1	18.61	5.11
G2	17.88	2.56
G3	28.83	2.56
G4	20.44	4.01

Table 7: TET2 (p-value = 4.2230E-01)

	No mutation (in %)	Mutation (in %)
G1	18.61	5.11
G2	14.60	5.84
G3	23.72	7.66
G4	20.44	4.01

Table 8: FLT3 mutation (p-value = 1.9160E-06)

	No mutation (in %)	Mutation (in %)
G1	18.25	5.47
G2	14.96	5.47
G3	16.42	14.96
G4	22.26	2.19

Table 9: FLT3-ITD (p-value = 2.8758E-06)

	No duplication (in %)	Duplication (in %)
G1	18.25	5.47
G2	16.06	4.38
G3	17.88	13.50
G4	22.99	1.46

Table 10: Karyotype (p-value = 3.9627E-03)

	Abnormal (in %)	Normal (in %)
G1	9.96	13.79
G2	9.20	11.88
G3	14.56	17.24
G4	16.48	6.90

Table 11: t(8;21) (p-value = 2.3159E-01)

	Abnormal (in %)	Normal (in %)
G1	0.00	23.75
G2	0.38	20.69
G3	0.38	31.42
G4	1.15	22.22

Table 12: inv(16)/t(16;16) (p-value = 9.0495E-03)

	Abnormal (in %)	Normal (in %)
G1	0.00	23.75
G2	0.77	20.31
G3	0.38	31.42
G4	2.30	21.07

Table 13: t(15;17) (p-value = 6.5962E-01)

	Abnormal (in %)	Normal (in %)
G1	1.15	22.61
G2	0.38	20.69
G3	1.53	30.27
G4	0.38	22.99

Table 14: inv(3)/t(3;3) (p-value = 8.6716E-01)

	Abnormal (in %)	Normal (in %)
G1	0.38	23.37
G2	0.38	20.69
G3	0.38	31.42
G4	0.77	22.61

Table 15: t(11q23) (p-value = 2.9389E-01)

	Abnormal (in %)	Normal (in %)
G1	0.00	23.75
G2	1.15	19.92
G3	0.77	31.03
G4	0.77	22.61

Table 16: del5 (p-value = 7.8496E-03)

	Abnormal (in %)	Normal (in %)
G1	1.92	21.84
G2	0.77	20.31
G3	0.38	31.42
G4	3.45	19.92

Table 17: del7 (p-value = 1.5314E-02)

	Abnormal (in %)	Normal (in %)
G1	2.68	21.07
G2	0.38	20.69
G3	2.68	29.12
G4	4.60	18.77

Table 18: +8 (p-value = 2.4024E-01)

	Abnormal (in %)	Normal (in %)
G1	0.77	22.99
G2	1.92	19.16
G3	1.53	30.27
G4	2.68	20.69

Table 19: ELN Risk (p-value = 2.1069E-01)

	High (in %)	Intermediate (in %)	Low (in %)
G1	6.15	14.23	3.46
G2	3.85	12.31	5.00
G3	6.15	20.39	5.38
G4	8.08	10.38	4.62

Table 20: Etiology (p-value = 1.5194E-01)

	de-novo (in %)	s-AML (in %)	t-AML (in %)
G1	17.65	1.47	4.04
G2	16.54	1.47	2.57
G3	26.84	2.94	1.84
G4	20.59	2.94	1.10

Table 21: lncRNA vs. mRNA clusters (p-value = 2.5615E-68)

	C1 (in %)	C2 (in %)	C3 (in %)	C4 (in %)	C5 (in %)	C6 (in %)	C7 (in %)
G1	0.00	13.87	1.46	5.84	0.36	1.46	0.73
G2	0.00	1.09	0.00	9.49	4.38	0.36	5.11
G3	8.76	0.00	5.11	0.00	1.46	16.06	0.00
G4	0.36	0.00	9.12	0.00	5.84	1.82	7.30
