# Supplementary material for: Expression levels of long non-coding RNAs are prognostic for AML outcome
Source: J Hematol Oncol. 2018 Apr 7;11:52. doi: 10.1186/s13045-018-0596-2 (PMC5889529; doi:10.1186/s13045-018-0596-2)
Supplement: Supplementary file 5 — Supplementary methods. Description of data: Detailed description of the methods used in the analysis. (DOCX 66 kb) [file 13045_2018_596_MOESM5_ESM.docx]

**Supplementary Material and Method**

**Title:** Expression levels of long non-coding RNAs are prognostic for AML outcome

**Authors:** Arvind Singh Mer^1§^, Johan Lindberg^2§^, Christer Nilsson^3^, Daniel Klevebring^1^, Mei Wang^1^, Henrik Grönberg^1^, Sören Lehmann^3,4^, Mattias Rantalainen^1*^

**Author affiliation:**

^1^ Department of Medical Epidemiology and Biostatistics, Karolinska Institutet, Nobels Vag 12A, SE-17177 Stockholm, Sweden

^2^ Department of Medical Epidemiology and Biostatistics, Science for Life Laboratory, Karolinska Institutet, Nobels Vag 12A, SE-17177 Stockholm, Sweden

^3^ Hematology Centre, Karolinska University Hospital and Karolinska Institute, Huddinge, Stockholm Sweden

^4^ Department of Medical Sciences, Uppsala University, Uppsala, Sweden

§ These authors contributed equally to this work

* Corresponding author

**Materials and methods**

**Clinseq cohort**

This study was approved by the regional ethical review board in Stockholm, Sweden. AML patient samples were obtained from the patients in the Clinseq-AML cohort. All patients in the Clinseq-AML cohort were treated with intensive induction regimens, including anthracyclines and cytosine arabinoside, according to Swedish national guidelines [1], between February 1997 and August 2014. Bone marrow or peripheral blood samples from the patients were collected at the time of diagnosis and separated for mononuclear cells. All samples were stored in isothermal liquid nitrogen freezers at -180 degrees Celsius until analysis. Clinical data was retrieved from the patient records and the Swedish Adult Acute Leukemia Registry [2]. For detail characteristics of Clinseq-AML cohort see Table-1 in the main manuscript.

**TCGA AML cohort**

For validation we utilised The Cancer Genome Atlas (TCGA) AML cohort which includes 142 AML patients with intensive induction treatment [3]. Clinical and mutational data was retrieved from the data portal of TCGA (https://tcga-data.nci.nih.gov/tcga/) and Table S1 of the publication of the TCGA AML study. The median follow-up time in TCGA cohort was 346.5 days. The detail characteristics of the patients in TCGA-AML cohort were described in their original publication [3].

**Sample preparation and sequencing**

To construct the DNA libraries ThruPlex-FD (Rubicon Genomics) was used. We constructed two aliquots, one used for low-pass whole genome sequencing (WGS) and the other aliquot was used for sequence capture[4] using a custom-made pan-cancer panel. The panel contains 655 genes, identified through an extensive literature search. Both genes associated with cancer through somatic mutations and increased germline risk was included. Samples were sequenced using the Illumina HiSeq 2500 platform. Low-pass WGS was performed to a depth of 0.5X coverage allowing for identification of copy-number alterations [5]. The panel was sequenced to ~360X coverage for identification of point mutations and indels. Ribosomal RNA depletion was performed using the Ribo-Zero gold kit, containing probes targeting both cytoplasmic and mitochondrial ribosomal RNA. The TruSeq Stranded Total RNA library prep kit was used to prepare the depleted RNA for sequencing. The RNA-seq libraries were sequenced to an average depth of 33 million read-pairs per library (paired-end 2 x 101 bases). For the pre-processing AutoSeq (https://github.com/clinseq/autoseq) was used, which includes best practices pipelines for the sequencing data.

**Bioinformatic processing of RNA sequencing data**

Using skewer version 0.1.117[6] standard Illumina adapters were trimmed. Alignment was performed using STAR aligner version 2.4.0e[7]. PCR duplicates were marked but not removed, using Picard MarkDuplicates version 1.128 (http://broadinstitute.github.io/picard). HTSeq count version 0.6.1[8] was used for gene expression estimation. Genes with zero count were excluded. RNAseq count data normalization was performed using the TMM method [9] in edgeR package[10]. From the expression data, we selected all probes for which read count per million is more than one in 10% of samples. A total of 3030 lncRNAs were annotated using MiTranscriptome database [11].

**Subtype discovery analysis**

For subtype discovery consensus clustering based unsupervised learning approach was applied [12]. From the lncRNA expression profile containing 274 samples and 3030 features, 80% of samples and 80% of the features were randomly selected. We applied the K-medoids clustering algorithm on this dataset, where the value of k (number of clusters) varies from 2 to 8 clusters. During initialisation, the algorithm randomly selects k data-points as the medoids and assigns other data points to its nearest medoid. We utilised Spearman's rank correlation as the distance measure between the medoids and the data-point. In the next iteration medoids are calculated for each cluster and the data points are reassigned to clusters based on distance from new medoide. The algorithm runs iteratively and tries to minimise the error function until it converges. The cluster assigned to each sample is recorded. This procedure is repeated 1000 times by randomly selecting 80% samples and 80% of the features from the complete dataset. We aggregated the results from each round and created consensus matrices. For each k, samples were assigned to a cluster based on the consensus matrices. Figure S1 shows the consensus matrices at k=2 to 8.

To find the optimal number of clusters, we utilised a weighted silhouette approach. For a data point in a cluster, the silhouette value is a measure of how similar the point is to points in its own cluster, when compared to points in other clusters. Thus a higher silhouette value indicates that the clusters are well separated. At k=4 the silhouette width is highest which indicated the presence of four clusters in the dataset (Supplementary Figure S2). Furthermore we calculated the Ratkowsky-Lance index[13] which also indicated that four is the optimal number of clusters in the dataset (Supplementary Figure S3).

**Validation of lncRNA subtype using cross-validation**

We applied 10-fold cross-validation to validate the lncRNA subtypes. At each cross-validation round, lncRNA expression data was randomly divided into a train set and test set. The train set was used for unsupervised subtype discovery as described earlier. In each cross-validation round unsupervised learning was performed. Label switching during the cross-validation rounds was addressed by comparing the centroid of each cluster to the centroid of clusters using complete data and assign group labels to their nearest centroid. Next, the training data and cluster labels from the unsupervised learning were utilized for supervised learning (using random forest [14]) and the test dataset labels were predicted. Predicted labels from the 10-fold cross-validation procedure were aggregated and the prognostic performances of the subtypes were assessed using survival analysis.

**Validation of lncRNA subtype in an external cohort**

The lncRNA expression based subtypes were validated in the independent TCGA AML patient cohort. TCGA AML RNAseq and clinical data was downloaded from the TCGA AML data portal, in accordance with the TCGA data access and publication guidelines. Raw data was processed using the pipeline described above. The TCGA cohort has RNAseq libraries prepared using poly-A selection, leading to fewer lncRNA molecules being quantified compared to the Clinseq cohort. We found that 2616 lncRNA were present in both Clinseq and TCGA lncRNA expression dataset. We used the subset expression data from both cohorts for these common features and applied Combat [15] to remove the batch effect. Using the random forest algorithm [14] supervised learning method we trained classifier on batch corrected Clinseq AML lncRNA expression data, with subtype labels assigned as described above. During the training process optimized number of trees using 10-fold internal cross validations and class balance accuracy as performance matrix. The optimized classifier was used to predict the subtype for TCGA AML samples, and enabled subsequent survival analysis.

**Messenger RNA expression data clustering**

We applied the consensus clustering protocol similar to lncRNA expression based subtype discovery for mRNA expression data clustering. From the raw read count RNA sequencing data, we selected all the annotated mRNA. In the mRNA expression data, features which have a read count equal to zero in more than 10% of samples were removed. Raw read count data was normalised using “Deseq” method [16]. Consensus clustering was applied to discover the robust cluster where the number of clusters (k) varies from 2 to 8 clusters. For each value of k, we performed 1000 fold Monte Carlo sub-sampling on both samples and features. By aggregating clustering labels from different sub-sampling rounds, consensus matrices were created. Samples were assigned clusters based on consensus matrices. Similar to lncRNA subtype discover protocol, weighted silhouette approach was used for finding optimal number of clusters in mRNA expression dataset.

**Molecular and clinical association analysis**

The distribution of molecular and clinical data by the lncRNA based consensus clusters is shown in Figure 1. We performed chi-square test for association analyses and p-values were corrected for multiple testing using the Benjamini-Hochberg method [17]. All analyses were carried out using R statistical software version 3.1.1. For mutation profiling, targeted sequencing was performed on frequently mutated exons in AML such as *CEBPA*, *NPM1*, *TP53* and *FLT3*.

**Survival analysis**

We measured overall survival from the date of diagnosis of AML to the date of death. Patients who were alive at last follow-up were censored. Survival outcomes were estimated using Kaplan-Meier curve and non-parametric log-rank statistic was used for comparison of the groups. Uni-variable and multivariable Cox's proportional hazards regression models were fitted to the follow up data to obtain the hazard ratios and 95% confidence intervals (CI). In multivariate Cox’s proportional hazard model we adjusted for age (dichotomized at 60 years), sex, etiology (de novo, secondary or therapy-related AML), ELN score, mutational status of NPM1, FLT3-ITD and CEBPA, TP53, WT1, TET2, ASXL1, DNMT3A, RUNX1, IDH1, IDH2 and cytogenetic aberrations including normal karyotype, inv(16)/t(16;16), del5, del7, t(8;21), +8, t(11q23), t(15;17), inv(3)/t(3;3). Analysis was carried out using R (version 3.1.1).

**Pathway analysis**

Differentially expressed genes (mRNA) were determined from RNA-seq data as those with differential expression levels between either of the groups of patients defined by the four lncRNA expression-based subtypes. Using the *limma/voom* R package for RNA-seq data analysis [18], read count data were transformed to log-counts. Empirical Bayes moderated t-statistics was applied to analyse differential expressed genes, using functions in the R package limma [19]. The Benjamini and Hochberg false discovery rate (FDR) was used to adjust for multiple testing [17]. Differentially expressed genes were defined as those with FDR-adjusted p-value<0.01. A pathway enrichment analysis of the set of differentially expressed genes was conducted using the MSigDB database [20] and pathway overrepresentation was tested by a hypergeometric model [21].

**Reference**

1. Wahlin A, Billstrom R, Bjor O, Ahlgren T, Hedenus M, Hoglund M, Lindmark A, Markevarn B, Nilsson B, Sallerfors B, Brune M: **Results of risk-adapted therapy in acute myeloid leukaemia. A long-term population-based follow-up study.** *European Journal of Haematology* 2009, **83:**99-107.

2. Lazarevic V, Horstedt AS, Johansson B, Antunovic P, Billstrom R, Derolf A, Hulegardh E, Lehmann S, Mollgard L, Nilsson C, et al: **Incidence and prognostic significance of karyotypic subgroups in older patients with acute myeloid leukemia: the Swedish population-based experience.** *Blood Cancer J* 2014, **4:**e188.

3. Cancer Genome Atlas Research N: **Genomic and epigenomic landscapes of adult de novo acute myeloid leukemia.** *N Engl J Med* 2013, **368:**2059-2074.

4. Lindberg J, Klevebring D, Liu W, Neiman M, Xu J, Wiklund P, Wiklund F, Mills IG, Egevad L, Gronberg H: **Exome sequencing of prostate cancer supports the hypothesis of independent tumour origins.** *Eur Urol* 2013, **63:**347-353.

5. Scheinin I, Sie D, Bengtsson H, van de Wiel MA, Olshen AB, van Thuijl HF, van Essen HF, Eijk PP, Rustenburg F, Meijer GA, et al: **DNA copy number analysis of fresh and formalin-fixed specimens by shallow whole-genome sequencing with identification and exclusion of problematic regions in the genome assembly.** *Genome Res* 2014, **24:**2022-2032.

6. Jiang H, Lei R, Ding SW, Zhu S: **Skewer: a fast and accurate adapter trimmer for next-generation sequencing paired-end reads.** *BMC Bioinformatics* 2014, **15:**182.

7. Dobin A, Davis CA, Schlesinger F, Drenkow J, Zaleski C, Jha S, Batut P, Chaisson M, Gingeras TR: **STAR: ultrafast universal RNA-seq aligner.** *Bioinformatics* 2013, **29:**15-21.

8. Anders S, Pyl PT, Huber W: **HTSeq--a Python framework to work with high-throughput sequencing data.** *Bioinformatics* 2015, **31:**166-169.

9. Robinson MD, Oshlack A: **A scaling normalization method for differential expression analysis of RNA-seq data.** *Genome Biol* 2010, **11:**R25.

10. Robinson MD, McCarthy DJ, Smyth GK: **edgeR: a Bioconductor package for differential expression analysis of digital gene expression data.** *Bioinformatics* 2010, **26:**139-140.

11. Iyer MK, Niknafs YS, Malik R, Singhal U, Sahu A, Hosono Y, Barrette TR, Prensner JR, Evans JR, Zhao S, et al: **The landscape of long noncoding RNAs in the human transcriptome.** *Nat Genet* 2015, **47:**199-208.

12. Wilkerson MD, Hayes DN: **ConsensusClusterPlus: a class discovery tool with confidence assessments and item tracking.** *Bioinformatics* 2010, **26:**1572-1573.

13. Ratkowsky D, Lance G: **A criterion for determining the number of groups in a classification.** *Australian Computer Journal* 1978, **10:**115-117.

14. Breiman L: **Random Forests.** *Machine Learning* 2001, **45:**5-32.

15. Johnson WE, Li C, Rabinovic A: **Adjusting batch effects in microarray expression data using empirical Bayes methods.** *Biostatistics* 2007, **8:**118-127.

16. Anders S, Huber W: **Differential expression analysis for sequence count data.** *Genome Biol* 2010, **11:**R106.

17. Benjamini Y, Hochberg Y: **Controlling the false discovery rate: a practical and powerful approach to multiple testing.** *Journal of the royal statistical society Series B (Methodological)* 1995**:**289-300.

18. Law CW, Chen Y, Shi W, Smyth GK: **voom: Precision weights unlock linear model analysis tools for RNA-seq read counts.** *Genome Biol* 2014, **15:**R29.

19. Ritchie ME, Phipson B, Wu D, Hu Y, Law CW, Shi W, Smyth GK: **limma powers differential expression analyses for RNA-sequencing and microarray studies.** *Nucleic Acids Res* 2015, **43:**e47.

20. Liberzon A, Subramanian A, Pinchback R, Thorvaldsdottir H, Tamayo P, Mesirov JP: **Molecular signatures database (MSigDB) 3.0.** *Bioinformatics* 2011, **27:**1739-1740.

21. Boyle EI, Weng S, Gollub J, Jin H, Botstein D, Cherry JM, Sherlock G: **GO::TermFinder--open source software for accessing Gene Ontology information and finding significantly enriched Gene Ontology terms associated with a list of genes.** *Bioinformatics* 2004, **20:**3710-3715.
