# Supplementary material for: Expression levels of long non-coding RNAs are prognostic for AML outcome
Source: J Hematol Oncol. 2018 Apr 7;11:52. doi: 10.1186/s13045-018-0596-2 (PMC5889529; doi:10.1186/s13045-018-0596-2)
Supplement: Supplementary file 7 — Description of TCGA-AML cohort. Description of data: Clinical characteristics of the TCGA-AML cohort used in this study. (PDF 46 kb) [file 13045_2018_596_MOESM7_ESM.pdf]

	TCGA (N=142)
**Sex: No. of male (%)**	74 (52.1%)
**Age: median (range)**	53 (18-81)
**AML etiology type**	
- **De novo AML**	142
- **s-AML**	0
- **t-AML**	0
- **Missing**	0
**Median follow-up (days)**	455.5
**Bone marrow blast: median (range, %)**	75% (30-100%)
**White blood cell counts: median (range, per mm^3)**	21.1 (0.4-297.4)
**Cytogenetic aberrations: N (%)**	
- **normal**	64 (45.1%)
- **complex**	19 (13.4%)
- **t(15;17)**	13 (9.2%)
- **t(8;21)**	7 (4.9%)
- **inv(16)/t(16;16)**	11 (7.7%)
- **inv(3)/t(3;3)**	0
- **del(5)**	8 (5.6%)
- **del(7)**	14 (9.9%)
- **t(11q23)**	4 (2.8%)
**Mutation: N (%)**	
- **ASXL1**	2 (1.41%)
- **CEBPA**	12 (8.45%)
- **CEBPA (double)**	5 (3.52%)
- **DNMT3A**	34 (23.94%)
- **FLT3-TKD**	38 (26.76%)
- **FLT3-ITD**	29 (20.42%)
- **IDH1**	15 (10.56%)
- **IDH2**	13 (9.15%)
- **KRAS**	3 (2.11%)
- **NPM1**	40 (28.17%)
- **RUNX1**	11 (7.75%)
- **TET2**	11 (7.75%)
- **TP53**	9 (6.34%)
- **WT1**	9 (6.34%)

Description of TCGA-AML cohort. Abbreviations: WBC, white blood cell; ELN, European LeukemiaNet; FLT3-ITD, internal tandem duplication of the FLT3 gene; FLT3-TKD, tyrosine kinase domain mutation in the FLT3 gene; t-AML, therapy-related acute myeloid leukemia; s-AML, secondary acute myeloid leukemia
